# Aquifer-Mediated Speciation in Cave-Adapted Fishes

**DOI:** 10.1093/iob/obag021

**Published:** 2026-05-18

**Authors:** C D Brownstein, G J Watkins-Colwell, M Policarpo, R C Harrington, E A Hoffman, D Casane, T J Near

**Affiliations:** Department of Ecology and Evolutionary Biology, Yale University, New Haven CT, USA; Yale Peabody Museum, New Haven CT, USA; Zoological Institute, Department of Environmental Sciences, University of Basel, Basel, Switzerland; Evolution of Sensory and Physiological Systems, Max Planck Institute for Biological Intelligence, Martinsried, Germany; Marine Resources Research Institute, South Carolina Department of Natural Resources; Richard Gilder Graduate School, American Museum of Natural History, NY, NY, USA; Université Paris-Saclay, CNRS, IRD, UMR Évolution, Génomes, Comportement et Écologie, 91198 Gif-sur-Yvette, France; Université Paris Cité, UFR Sciences du Vivant, F-75013 Paris, France; Department of Ecology and Evolutionary Biology, Yale University, New Haven CT, USA; Yale Peabody Museum, New Haven CT, USA

## Abstract

The nature of speciation within subterranean ecosystems following invasions from the surface remains poorly understood. Most proposed examples of *in situ* subterranean speciation instead appear to reflect multiple independent surface invasions, supporting the classic hypothesis that subterranean ecosystems are evolutionary dead ends. Here, we examine the species diversity within the most widespread subterranean vertebrate species, the Southern Cavefish *Typhlichthys subterraneus*. Phylogenomic analyses reveal that *T. subterraneus* as currently recognized is paraphyletic with respect to the Missouri Cavefish *T. eigenmanni*, as a distinct set of populations is resolved as the sister lineage of a clade formed by *T. eigenmanni* and *T. subterraneus sensu stricto*. High-resolution computed tomography (CT) scanning reveals skeletal autapomorphies of this lineage, supporting its recognition as a new species: *Typhlichthys styx* sp. nov. Ancestral biogeographic reconstructions reveal that speciation in *Typhlichthys* has occurred along aquifer boundaries, with lineages dispersing through widespread karstic aquifer systems across southeastern and central North America. This dispersal facilitated secondary sympatry among cavefish species that last shared common ancestry approximately eight million years ago. Together, these results reveal aquifer geology as a driver of allopatric speciation in obligate cave-dwelling vertebrates, with implications for understanding biodiversity in subterranean ecosystems worldwide.

## Introduction

Speciation is the central process in which biodiversity is generated (Mayr 1947; Bush 1975; Turelli et al. 2001; Gavrilets 2003; Coyne and Orr 2004), yet it remains debated whether different ecosystems tend to facilitate different mechanisms of speciation (Wilson and Hessler 1987; Coyne and Orr 2004; Milks et al. 2024). Environments characterized by extreme physiochemical conditions and resource scarcity, such as the deep sea (Wilson and Hessler 1987; Palumbi 1994; Angel 1997; Laakmann et al. 2012; Davis et al. 2014; Eilertsen and Malaquias 2015), caves (Poulson and White 1969; Barr and Holsinger 1985; Howarth 1993; Finston et al. 2007; Ribera et al. 2010; Bloom et al. 2014; Culver and Pipan 2019; Devitt et al. 2019; Mammola 2019; Li et al. 2020; Langille et al. 2021; Aharon et al. 2023; Milks et al. 2024), and geochemically influenced springs (Tobler, Scharnweber, et al. 2015, et al. 2015; Tobler et al. 2018) have often been the focus of biologists investigating the existence and prevalence of alternative modes of speciation to lineage divergence induced by geographic isolation, which is known as allopatry. This line of research is driven by the observation that in many of these ecosystems, closely related species appear to exist in sympatry or lack population structure defined by geographic regions (Wilson and Hessler 1987; Palumbi 1994; Niemiller et al. 2012; Niemiller, Graening, et al. 2013; Davis et al. 2014; Li et al. 2020; Hart et al. 2024).

Although underground environments contain an exceptionally high proportion of endemic biodiversity (Poulson and White 1969; Culver and Pipan 2019; Mammola 2019; Niemiller and Taylor 2019; Milks et al. 2024), whether speciation has occurred in subterranean ecosystems among organisms obligately adapted to them remains controversial. Analyses of molecular data have shown that nearly all obligate subterranean species have independently colonized these ecosystems, even when their closest relatives also only live underground (Wiens et al. 2003; Niemiller et al. 2012; Devitt et al. 2019; Langille et al. 2021; Mao et al. 2021; Mao et al. 2022; Aharon et al. 2023; Benito et al. 2025; Brownstein et al. 2025; Niida et al. 2025). Lineages comprised of high proportions of obligate subterranean species appear to have invaded underground environments multiple times and often include facultatively subterranean forms that may approximate the ancestors of independently evolved obligate cave species (Niemiller et al. 2008; Niemiller, Fitzpatrick, et al. 2013; Devitt et al. 2019; Aharon et al. 2023; Benito et al. 2025; Brownstein et al. 2025). In cases where obligate subterranean species are sister taxa, examinations of the molecular basis of morphological and physiological features, such as the loss of functional eyes, that are tied to obligate cave dwelling demonstrate that sister species have convergently lost these features rather than inherited them from a single common ancestor (Niemiller, Fitzpatrick, et al. 2013; Casane and Rétaux 2016; Fumey et al. 2018; Herman et al. 2018). This means that there are surprisingly few cases of *in situ* subterranean speciation (Faille et al. 2010; Niemiller et al. 2012; Hadid et al. 2013; Niemiller, McCandless, et al. 2013; Cieslak et al. 2014; Li et al. 2015; Langille et al. 2021; Hart et al. 2024). Almost all of these examples of *in situ* subterranean speciation involve very young sister species pairs (Niemiller, McCandless, et al. 2013; Langille et al. 2021) and invoke controversial mechanisms such as sympatric speciation in the absence of obvious geographic isolation (Hadid et al. 2013; Li et al. 2015; Li et al. 2020).

Our poor understanding of subterranean speciation both inhibits the assessment and protection of underground biodiversity (Wiens et al. 2003; Niemiller et al. 2012; Niemiller, Graening, et al. 2013; Devitt et al. 2019; Langille et al. 2021; Hart et al. 2024) and impedes our knowledge of the assembly and maintenance of functionally diverse subterranean faunas (Camacho 1992; Danielopol et al. 2000; Gibert and Deharveng 2002; Culver and Pipan 2019; Milks et al. 2024) that face unique threats as anthropogenic activities deplete and degrade groundwater resources (Danielopol et al. 2000; Foster and Chilton 2003; Aeschbach-Hertig and Gleeson 2012; Famiglietti 2014; Bierkens and Wada 2019; Devitt et al. 2019; Jurgens et al. 2022; Jasechko et al. 2024; Saccò et al. 2024).

The rarity of subterranean speciation might be taken as evidence for the hypothesis that subterranean ecosystems are evolutionary dead ends (Darwin 1859; Poulson and White 1969; Barr and Holsinger 1985; Culver and Pipan 2019; Mammola 2019), yet the existence of subterranean species comprised of geographically isolated, deeply divergent sub-lineages that may themselves be consistent with species under a general lineage species concept (De Queiroz 2007), including several from the massive underground karst ecosystems of eastern North America (Christman et al. 2005), suggests other explanations are possible (Niemiller et al. 2012; Stern et al. 2017; Hart et al. 2024). These ecosystems are home to the most widespread species of subterranean vertebrate, the Southern Cavefish *Typhlichthys subterraneus*, which occurs in karstic cave environments throughout the southeastern United States. Since the 1980s, phylogenetic analyses of genetic data have demonstrated that *Typhlichthys subterraneus* likely represents a complex of numerous ancient lineages (Swofford 1982; Niemiller et al. 2012; Niemiller, Graening, et al. 2013, et al. 2013; Hart et al. 2024; Brownstein et al. 2025), yet the delimitation and description of species in this lineage has remained difficult owing to the absence of known features that distinguish deeply divergent lineages in *Typhlichthys* phylogeny (Burress et al. 2017; Hart et al. 2024).

Here, by combining phylogenomics with high-resolution computed tomography, we delimit and describe species diversity in *Typhlichthys.* Our results allow us to recognize a new, wide-ranging third species in this genus and establish that the biogeography of *Typhlichthys* corresponds to distinctions between major aquifers in Paleozoic rocks within the Appalachian Plateau, Valley and Ridge, and Ozark Plateau physiographic regions. In particular, aquifers in Carboniferous-aged limestone-rich rock formations appear to have facilitated subterranean dispersal in lineages of *Typhlichthys* that diverged over 5 million years ago yet show few morphological differences. These results substantiate a potential mechanism of allopatric speciation in a phenotypically conserved obligate subterranean lineage.

## Methods

### Locality data

**Fig. 1:**
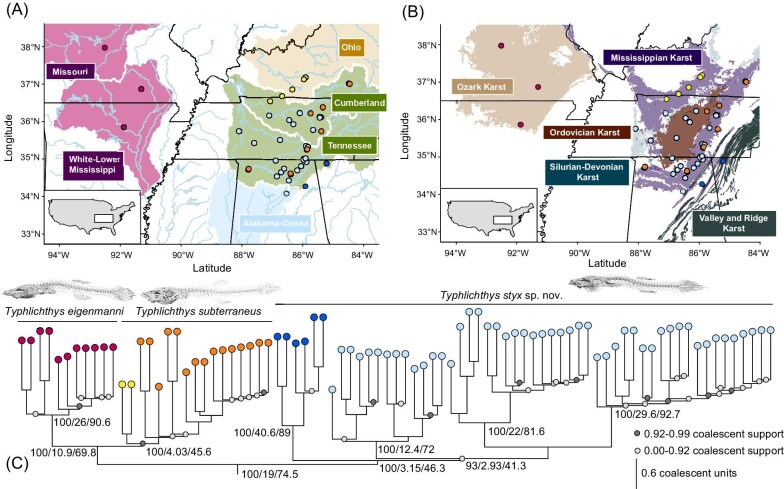
**Phylogeny and Biogeography of *Typhlichthys* Cavefishes.** Maps of the southeastern United States showing localities with available lat-long data sampled in our UCE and mtDNA phylogenies of *Typhlichthys* superimposed on (A) major drainages and (B) karstic formations (data from USGS). In A, major drainage basins are highlighted by taxon presence: purple indicates drainages inhabited by *Typhlichthys eigenmanni*, light blue indicates drainages inhabited by *T. styx*, orange indicates drainages inhabited by *T. subterraneus*, and green indicates drainages inhabited by both *T. styx* and *T. subterraneus*. Localities are colored by lineage. Major rivers and lakes are in light blue, and state borders are in black. In (B), the karstic formation extents are surface outcrops; bedrock outcrops of Mississippian rock in the region of Illinois appear more connected based on sinkhole distributions (Anon 2020). Phylogeny in (C) is the tree inferred under an ASTRAL-III multispecies coalescent framework from gene trees generated in IQ-TREE2. Numbers at nodes indicate bootstrap supports, gene, and site concordance factors, respectively, calculated for the same nodes (representing MRCAs of major lineages) in the IQ-TREE maximum likelihood phylogenetic and concordance factor analyses. Colored dots at nodes represent coalescent support values calculated in ASTRAL-III.

We obtained locality information for all specimens sequenced for ultraconserved elements and *ND2* mitochondrial DNA from a combination of previous literature (Niemiller et al. 2012; Niemiller, Graening, et al. 2013; Niemiller et al. 2016; Hart et al. 2020; Hart et al. 2024; Hart et al. 2025), museum databases (https://collections.peabody.yale.edu/; https://aumnh.auburn.edu/research-collections/vertebrate-zoology/fishes/search-fish-database/), and personal communication with M.L. Niemiller. Next, we acquired drainage data from Natural Earth (https://www.naturalearthdata.com/) for major North American river systems and lakes, river system border data from HydroBASINs (https://www.hydrosheds.org/products/hydrobasins), and principal US aquifer data from the United States Geological Survey (Anon 2021). Briefly, we define aquifers as water-holding underground geological structures and karst as a rock environment formed by the dissolution of soluble rock types, such as limestones and other carbonates. We noticed that the principal US aquifer data from the USGS was not fine-grained enough to reliably identify the karst aquifer association of each locality; many caves (i.e., Key Cave, Alabama) that would have been classified as Pennsylvanian sandstone associated actually are within small portions of Mississippian karst. To this end, we examined geological maps of the US states of Tennessee, Alabama, Georgia, and Kentucky available from the USGS State Geological Map Compilation (Horton et al. 2017), pulled names of relevant Cambrian through Mississippian limestone and dolomite units, and plotted their distributions using the R package *rmacrostrat* (Jones et al. 2024). We then checked to see whether the distribution of the set of formations plotted matched with previously published high-resolution maps of karst units in the United States (Weary and Doctor 2014). Finally, we plotted karst distributions in Kentucky using data from the Kentucky Geological Survey (https://www.uky.edu/KGS/karst/karst_resources.php) in order to assess for the possible connectivity of karst across the eastern and western Mississippian aquifers. For historical biogeographic analyses, we classified cavefish localities (Tables 1-4, Table 10) based on their association with particular aquifer systems (Fig. 1B). Because *Typhlichthys styx* sp. nov. is sympatric with *T. subterraneus* across many localities in Tennessee and Alabama corresponding to the eastern Mississippian aquifer, we could not include occurrences from major ichthyological locality data repositories, such as FishNet2. Complete sets of locality and lat-long coordinate data for specimens sequenced for UCEs and mtDNA are in Tables 2-3, and Table 4, respectively.

**Table 1:** Collection localities and museum catalog information for specimens used in morphological analyses. AUM, Auburn University Ichthyology Collection; UMMZ, University of Michigan Museum of Zoology Collection; YPM ICH, Ichthyology, Yale Peabody Museum, Yale University. Asterisks indicate specimens for which CT scans were taken or available. Bolded specimens are the hypodigm of the new species.

Species	Catalog number	Locality	Latitude	Longitude
*Typhlichthys eigenmanni*	AUM 57002*	Carroll Cave, MO	37.972044	−92.507179
*Typhlichthys eigenmanni*	UMMZ 156795*	Camden River Cave near Hahatonka, MO	–	–
*Typhlichthys eigenmanni*	UMMZ 150421*	Laclede, Bennett Spring source, Bennett State Park, MO	–	–
*Typhlichthys* sp.	AUM 58753*	Hering Cave, 4 mi NE Owen’s Crossroads, TN	34.62436	−86.40161
*Typhlichthys* sp.	AUM 56994*	Blowing Cave, near Cleghorn Creek, Falling Water River, TN	36.12611	−85.39833
*Typhlichthys* sp.	AUM 56995*	Jaco Spring Cave, Collins River-Upper Caney Fork, TN	35.78667	−85.66056
***Typhlichthys styx***	YPM ICH 25653.988*	**Flat Rock Cave (MLN12-41), Smith County, Cumberland, TN**	**36.235**	**−86.0992**
***Typhlichthys styx***	YPM ICH 25653.985*	**Flat Rock Cave (MLN12-41), Smith County, Cumberland TN**	**36.235**	**−86.0992**
***Typhlichthys styx***	YPM ICH 25653.989*	**Flat Rock Cave (MLN12-41), Smith County, Cumberland TN**	**36.235**	**−86.0992**
***Typhlichthys styx***	YPM ICH 25653.984*	**Flat Rock Cave (MLN12-41), Smith County, Cumberland TN**	**36.235**	**−86.0992**
*Typhlichthys styx*	YPM ICH 008000	Shelta Cave, 2 mi NW of Huntsville, Madison County, AL	34.754025	−86.61113
*Typhlichthys styx*	AUM 57001*	Baugus Cave, Decatur County, TN	35.7525	−88.08528
*Typhlichthys styx*	AUM 57001*	Baugus Cave, Decatur County, TN	35.7525	−88.08528
*Typhlichthys styx*	AUM 57001*	Baugus Cave, Decatur County, TN	35.7525	−88.08528
*Typhlichthys styx*	AUM 63167*	Limrock Blowing Cave, 10 mi NNW Scottsboro, AL	34.70889	−86.18139
*Typhlichthys styx*	AUM 56991*	Pattons Cave, Maury County, TN	36.05	−86.44667
*Typhlichthys styx*	AUM 57010*	Big Mouth Cave, Grundy County, TN	35.33278	−85.82694
*Typhlichthys styx*	AUM 58746*	Tally Ditch Cave, 7.9 km NNW Stevenson, AL	34.92944	−85.88083
*Typhlichthys styx*	YPM ICH 25594.0987*	Cave Stream (MLN 12–32), Garner Spring Cove, Crow Creek, TN	35.0286	−85.9072
*Typhlichthys styx*	YPM ICH 25594.0986*	Cave Stream (MLN 12–32), Garner Spring Cove, Crow Creek, TN	35.0286	−85.9072
*Typhlichthys subterraneus*	AUM 56984*	Bartlett Cave, near Martin Creek, TN	36.22806	−85.74361
*Typhlichthys subterraneus*	YPM ICH 25538.0995*	Dave’s Cave (MLN 12–015), Pulaski County, KY	37.0158	−84.485
*Typhlichthys subterraneus*	YPM ICH 25538.0996*	Dave’s Cave (MLN 12–015), Pulaski County, KY	37.0158	−84.485
*Typhlichthys subterraneus*	YPM ICH 25538.0997*	Dave’s Cave (MLN 12–015), Pulaski County, KY	37.0158	−84.485
*Typhlichthys subterraneus*	YPM ICH 25307.0992*	Webb Cave, Simpson County, KY	36.6786	−86.6772
*Typhlichthys subterraneus*	YPM ICH 25307.0993*	Webb Cave, Simpson County, KY	36.6786	−86.6772
*Typhlichthys subterraneus*	YPM ICH 25307.0994*	Webb Cave, Simpson County, KY	36.6786	−86.6772
*Typhlichthys subterraneus*	YPM ICH 25306. 0998*	Friendship Cave, Warren County, KY	36.8561	−86.3444
*Typhlichthys subterraneus*	YPM ICH 25306.999*	Friendship Cave, Warren County, KY	36.8561	−86.3444
*Typhlichthys subterraneus*	YPM ICH 25293.0990*	Drowned Rat Cave, Pulaski County, KY	37.0042	−84.4556
*Typhlichthys subterraneus*	YPM ICH 25293.0991*	Drowned Rat Cave, Pulaski County, KY	37.0042	−84.4556
*Typhlichthys subterraneus*	YPM ICH 25658.988*	Anderson Spring Cave (MLN 12–44), Putnam County, TN	36.1342	−85.4464
*Typhlichthys subterraneus*	YPM ICH 25658.989*	Anderson Spring Cave (MLN 12–44), Putnam County, TN	36.1342	−85.4464
*Typhlichthys subterraneus*	YPM ICH 20461*	Cave City, Barren County, KY	37.128017	−85.962423

**Table 2:** Biosample IDs and locality information for specimens for which UCE sequences were analyzed.

Biosample ID	Species	UCE Sample Number	Tissue Number	Locality	Drainage	County	State	Latitude	Longitude
SAMN20209475	*Amblyopsis hoosieri*	–	MLN 242	Donaldson Cave	Wabash	Lawrence	IN	38.4348	−86.2454
SAMN14308599	*Troglichthys rosae*	TrLOG01	L5	Logan Cave	Illinois	Benton	AR	36.1152	−94.2344
SAMN14308569	*Amblyopsis spelaea*	AsWEB03	YFTC 23,875	Webster Cave	Ohio	Breckinridge	KY	37.8847	−86.3547
SAMN14308566	*Aphredoderus sayanus*	Asay01	LSUMNS 3030/LSUMNZ 152,871	5 mi W Lucky	Mississippi	Bienville Parish	LA	–	–
SAMN14308576	*Chologaster cornuta*	Ccor08	NCSM 47,776	Unnamed tributary N. of Shaw Road at Bonnie Doone Lake	Cape Fear	Cumberland	NC	–	–
SAMN14308580	*Forbesichthys agassizii*	FaBLU02	UTTC 243	Blue Springs	Cumberland	DeKalb	TN	–	–
SAMN14308584	*Forbesichthys papilliferus*	FpKY02	YFTC 23,878	Spring-fed ditch N of crossing with Morton Rd	Red	Todd	KY	–	–
SAMN14308585	*Percopsis omiscomaycus*	PoIR01	TJN 237	Illinois River at Hennepin	Illinois	Putnam	IL	–	–
SAMN05915104	*Polymixia lowei*	–	YFTC 25,250	Bear Seamount, Atlantic Ocean	–	–	–	–	–
SAMN14308586	*Speoplatyrhinus poulsoni*	Sp01	UTTC 8233	Key Cave	Tennessee	Lauderdale	AL	34.7552	−87.7863
SAMN14308587	*Typhlichthys eigenmanni*	TeCAR01	MLN 416	Carroll Cave	Osage	Camden	MO	37.972044	−92.507179
SAMN14308588	*T. eigenmanni*	TeCAR02	MLN 425	Carroll Cave	Osage	Camden	MO	37.972044	−92.507179
SAMN14308589	*T. eigenmanni*	TeCON01	MLN 487	Concolor Cave	White	Howell	MO	–	–
SAMN14308590	*T. eigenmanni*	TeENN02	MLN EN2	Ennis Cave	White	Stone	AR	35.86278	−91.8675
SAMN14308591	*T. eigenmanni*	TeENN03	MLN EN3	Ennis Cave	White	Stone	AR	35.86278	−91.8675
SAMN14308592	*T. eigenmanni*	TeFAL01	MLN 398	Falling Spring Cave	White	Oregon	MO	36.868274	−91.294272
SAMN14308593	*T. eigenmanni*	TeMID01	YFTC23890	Midco Cave	White	Carter	MO	–	–
SAMN14308594	*T. eigenmanni*	TeNORF01	MLN NFL01	Norfolk Lake	White	Baxter	AR	–	–
SAMN14308595	*T. eigenmanni*	TePANT01	RIP 002	Panther Cave	White	Ripley	MO	–	–
SAMN14308596	*T. eigenmanni*	TePOSY01	MLN 426	Posy Spring Cave	White	Oregon	MO	–	–
SAMN14308597	*T. eigenmanni*	TeROAR01	MLN RS4	Roaring Spring Cave	White	Oregon	MO	–	–
SAMN14308600	*T. styx*	TsACC01	UTTC 04,822	Allens Creek Cave	Duck	Lewis	TN	35.4375	−87.59639
SAMN14308602	*T. subterraneus*	TsASC02	YFTC 26,817	Anderson Spring Cave	Cumberland	Putnam	TN	36.134167	−85.446390
SAMN14308603	*T. subterraneus*	TsBAR01	UTTC 04,824	Bartlett Cave	Cumberland	Putnam	TN	36.22806	−85.743610
SAMN14308604	*T. subterraneus*	TsBART01	CEW-16–04	Bartlett Cave	Cumberland	Putnam	TN	36.22806	−85.743610
SAMN14308605	*T. styx*	TsBAU01	MLN 293	Baugus Cave	Tennessee	Decatur	TN	35.752500	−88.085280
SAMN14308606	*T. styx*	TsBEE01	YFTC 26,838	Beech Spring Cave	Tennessee	Marshall	AL	34.438611	−86.458611
SAMN14308607	*T. styx*	TsBEE02	UAIC14821	Beech Spring Cave	Tennessee	Marshall	AL	34.438611	−86.458611
SAMN14308608	*T. styx*	TsBOB01	LSUMZ-F8641	Bobcat Cave	Tennessee	Madison	AL	34.655833	−86.711111
SAMN14308609	*T. styx*	TsBM01	UTTC04821	Big Mouth	Tennessee	Grundy	TN	35.332780	−85.826940
SAMN14308610	*T. styx*	TsBOB03	LSUMZ-F8643	Bobcat Cave	Tennessee	Madison	AL	34.655833	−86.711111
SAMN14308611	*T. styx*	TsBOB05	LSUMZ-F8645	Bobcat Cave	Tennessee	Madison	AL	34.655833	−86.711111
SAMN14308612	*T. styx*	TsBOB06	TSUB BC01	Bobcat Cave	Tennessee	Madison	AL	34.655833	−86.711111
SAMN14308613	*T. styx*	TsBS01	MLN 0030	Blowing Springs Cave	Tennessee	Coffee	TN	35.358802	−85.894192
SAMN14308614	*T. styx*	TsBS02	MLN 0031	Blowing Springs Cave	Tennessee	Coffee	TN	35.358802	−85.894192
SAMN14308615	*T. styx*	TsBS03	MLN 0033	Blowing Springs Cave	Tennessee	Coffee	TN	35.358802	−85.894192
SAMN14308616	*T. styx*	TsBS04	MLN 0050	Blowing Springs Cave	Tennessee	Coffee	TN	35.358802	−85.894192
SAMN14308617	*T. subterraneus*	TsCGC01	UTTC 04,815	Camps Gulf Cave no. 2	Cumberland	Van Buren	TN	35.751981	−85.391059
SAMN14308618	*T. styx*	TsCRA01	AUM 67212/LSUMNS	Crane Creek Cave	Tennessee	Catoosa	GA	34.89194	−85.21667
SAMN14308620	*T. styx*	TsCRY01	UTTC 04,812	Crystal Cave	Tennessee	Grundy	TN	35.27278	−85.85361
SAMN14308621	*T. styx*	TsCRY21	AUM 56984/LSUMNS	Crystal Cave	Tennessee	Grundy	TN	35.27278	−85.85361
SAMN14308622	*T. styx*	TsCSS01	ADCNR-A	Cave Spring Sink along Graves Creek	Mobile	Blount	AL	34.082569	−86.532984
SAMN14308623	*T. styx*	TsCSS02	ADCNR-B	Cave Spring Sink along Graves Creek	Mobile	Blount	AL	34.082569	−86.532984
SAMN14308624	*T. subterraneus*	TsDAV01	MLN 236	Dave’s Cave	Cumberland	Pulaski	KY	37.01536	−84.48557
SAMN14308625	*T. subterraneus*	TsDAV02	MLN 239	Dave’s Cave	Cumberland	Pulaski	KY	37.01536	−84.48557
SAMN14308626	*T. subterraneus*	TsEWSC01	UTTC 04,806	East Water Supply Cave	Cumberland	Overton	TN	36.38194	−85.33944
SAMN14308627	*T. styx*	TsFRC01	YFTC 26,795	Flat Rock Cave	Cumberland	Smith	TN	36.235	−86.09917
SAMN14308628	*T. styx*	TsFRC02	YFTC 26,796	Flat Rock Cave	Cumberland	Smith	TN	36.235	−86.09917
SAMN14308629	*T. styx*	TsGAL01	UTTC 04,808	South Gallagher Cave	Duck	Marshall	TN	35.51861	−86.74694
SAMN14308630	*T. styx*	TsGARN01	MLN GAR3	Garner Spring Cave	Tennessee	Franklin	TN	35.02861	−85.90722
SAMN14308631	*T. styx*	TsGARN02	MLN GAR4	Garner Spring Cave	Tennessee	Franklin	TN	35.02861	−85.90722
SAMN14308632	*T. styx*	TsGEI01	AUM 63191/LSUMNS	Geiger Cave	Tennessee	Jackson	AL	34.8207	−86.05331
SAMN14308633	*T. styx*	TsGEI02	AUM 63191/LSUMNS	Geiger Cave	Tennessee	Jackson	AL	34.8207	−86.05331
SAMN14308634	*T. styx*	TsGEI03	AUM 63191/LSUMNS	Geiger Cave	Tennessee	Jackson	AL	34.8207	−86.05331
SAMN14308635	*T. styx*	TsHER01	YFTC 26,839	Hering Cave	Tennessee	Madison	AL	34.62436	−86.40161
SAMN14308637	*T. subterraneus*	TsHER03	YFTC 26,841	Hering Cave	Tennessee	Madison	AL	34.62436	−86.40161
SAMN14308638	*T. styx*	TsHER13	LSUMNS	Hering Cave	Tennessee	Madison	AL	34.62436	−86.40161
SAMN14308639	*T. styx*	TsHER16	LSUMNS	Hering Cave	Tennessee	Madison	AL	34.62436	−86.40161
SAMN14308640	*T. styx*	TsHER17	LSUMNS	Hering Cave	Tennessee	Madison	AL	34.62436	−86.40161
SAMN14308641	*T. styx*	TsHERR01	UTTC 04,819	Herring Cave	Cumberland	Rutherford	TN	35.938610	−86.3075
SAMN14308642	*T. subterraneus*	TsHRC04	LSUMNS	Hidden River Cave	Ohio	Hart	KY	37.179209	−85.906629
SAMN14308643	*T. subterraneus*	TsJAQ01	MLN 0008	Jacques Cave	Cumberland	Putnam	TN	36.114170	−85.441390
SAMN14308644	*T. subterraneus*	TsJAQ02	MLN 0009	Jacques Cave	Cumberland	Putnam	TN	36.114170	−85.441390
SAMN14308645	*T. subterraneus*	TsJAQ03	MLN 0023	Jacques Cave	Cumberland	Putnam	TN	36.114170	−85.441390
SAMN14308646	*T. styx*	TsJAQ04	MLN 0024	Jacques Cave	Cumberland	Putnam	TN	36.114170	−85.441390
SAMN14308647	*T. subterraneus*	TsJSC01	MLN 0038	Jaco Spring Cave	Cumberland	Warren	TN	35.786670	−85.660560
SAMN14308648	*T. styx*	TsJSC02	MLN 0039	Jaco Spring Cave	Cumberland	Warren	TN	35.786670	−85.660560
SAMN14308649	*T. styx*	TsJSC03	MLN 0048	Jaco Spring Cave	Cumberland	Warren	TN	35.786670	−85.660560
SAMN14308650	*T. styx*	TsJSC04	MLN 0049	Jaco Spring Cave	Cumberland	Warren	TN	35.786670	−85.660560
SAMN14308651	*T. subterraneus*	TsKEY01	MLN KYC01	Key Cave	Tennessee	Lauderdale	AL	34.745556	−87.778889
SAMN14308652	*T. styx*	TsLBC01	AUF2602	Limrock Blowing Cave	Tennessee	Jackson	AL	34.70889	−86.18139
SAMN14308654	*T. styx*	TsLBC03	AUF2604	Limrock Blowing Cave	Tennessee	Jackson	AL	34.70889	−86.18139
SAMN14308655	*T. styx*	TsLCC01	MLN LCC01	Little Crow Creek Cave	Tennessee	Franklin	TN	35.0125	−85.96667
SAMN14308656	*T. styx*	TsLCC02	MLN LCC02	Little Crow Creek Cave	Tennessee	Franklin	TN	35.0125	−85.96667
SAMN14308657	*T. styx*	TsLRW01	MLN LRW2	Long’s Rock Wall	Tennessee	Dade	GA	–	–
SAMN14308658	*T. styx*	TsLRW01	MLN LRW2	Long’s Rock Wall	Tennessee	Dade	GA	–	–
SAMN14308659	*T. styx*	TsMKP01	MLN MKN1	McKinney Pit	Tennessee	Colbert	AL	34.725	−87.79111
SAMN14308660	*T. styx*	TsMKP02	MLN MKN2	McKinney Pit	Tennessee	Colbert	AL	34.725	−87.79111
SAMN14308661	*T. styx*	TsPAT01	MLN PT2	Pattons Cave	Cumberland	Rutherford	TN	36.05	−86.44667
SAMN14308662	*T. styx*	TsPAT02	MLN PT4	Pattons Cave	Cumberland	Rutherford	TN	36.05	−86.44667
SAMN14308663	*T. styx*	TsSEL01	JWA405	Sells Cave	Mobile	DeKalb	AL	34.27472	−85.91056
SAMN14308664	*T. styx*	TsSHL01	MLN SHL2	Shelta Cave	Tennessee	Madison	AL	34.754025	−86.61113
SAMN14308665	*T. styx*	TsSHL02	MLN SHL3	Shelta Cave	Tennessee	Madison	AL	34.754025	−86.61113
SAMN14308666	*T. subterraneus*	TsSINK01	MN-CP-JAC1	Sinking Ridge Cave	Cumberland	Robertson	TN	36.542806	−87.073138
SAMN14308667	*T. styx*	TsSRC01	MLN SAR5	Salt River Cave	Tennessee	Jackson	AL	34.988330	−85.97556
SAMN14308668	*T. styx*	TsSRC02	MLN SAR6	Salt River Cave	Tennessee	Jackson	AL	34.988330	−85.97556
SAMN14308669	*T. subterraneus*	TsSTA01	MLN 0290	Stamps Cave	Cumberland	Putnam	TN	36.12611	−85.39833
SAMN14308670	*T. subterraneus*	TsSTA02	MLN 0331	Stamps Cave	Cumberland	Putnam	TN	36.12611	−85.39833
SAMN14308671	*T. styx*	TsTC01	13–048	Twin Cave	Cumberland	Cheatham	TN	36.170211	−87.103513
SAMN14308672	*T. styx*	TsTDC01	YFTC 26,831	Tally Ditch Cave	Tennessee	Jackson	AL	34.92944	−85.88083
SAMN14308673	*T. styx*	TsTRU01	UTTC 04,820	Trussell Cave	Tennessee	Grundy	TN	35.25861	−85.87083
SAMN14308674	*T. styx*	TsWELL01	UAIC 14,842.01	Well in Morgan County, AL	Tennessee	Morgan	AL	34.546111	−86.851111
SAMN14308675	*T. styx*	TsWSC01	MLN-WSP1	White’s Spring Cave	Tennessee	Limestone	AL	34.967405	−86.890255

**Table 3:** Biosample IDs and summary statistics for sequences used in phylogenetic analyses of ultraconserved elements. *Abbreviations*: BP, base pairs, kb, kilobase pairs.

Biosample ID	Species	Contigs	Total BP	Mean Length	95% CI Length	Minimum Length	Maximum Length	Median Length	Contigs > 1kb
SAMN20209475	*Amblyopsis hoosieri*	752	845,513	1124.3523936170213	2.9353296307882757	526	1215	1133	713
SAMN14308599	*Troglichthys rosae*	918	963,374	1049.4270152505446	8.932354071659649	207	1989	1094	590
SAMN14308569	*Amblyopsis spelaea*	949	1,094,013	1152.8061116965227	9.834496086597571	57	2306	1170	693
SAMN14308566	*Aphredoderus sayanus*	951	1,030,071	1083.1451104100947	10.108805597386292	175	1865	1137	621
SAMN14308576	*Chologaster cornuta*	884	445,761	504.25452488687785	5.40016443	207	1017	503	2
SAMN14308580	*Forbesichthys agassizii*	937	914,407	975.8879402347919	8.513479679557324	207	1910	1012	484
SAMN14308584	*Forbesichthys papilliferus*	959	771,684	804.6757038581856	7.551713339017929	218	1545	812	194
SAMN14308585	*Percopsis omiscomaycus*	970	876,601	903.7123711340206	7.311886214222083	242	1803	928	364
SAMN05915104	*Polymixia lowei*	1017	628,883	618.370698	6.301493733923427	201	1213	612	29
SAMN14308586	*Speoplatyrhinus poulsoni*	567	168,538	297.24514991181655	2.988167421731974	104	686	283	0
SAMN14308587	*Typhlichthys eigenmanni*	947	840,237	887.2618796198522	8.263996621006305	159	1972	910	332
SAMN14308588	*T. eigenmanni*	924	839,147	908.1677489177489	8.874233041292392	141	1663	924	367
SAMN14308589	*T. eigenmanni*	955	736,997	771.7246073298429	7.414370482255155	193	1578	780	142
SAMN14308590	*T. eigenmanni*	946	884,699	935.1997885835095	7.131131831497943	213	1665	953.5	388
SAMN14308591	*T. eigenmanni*	950	914,062	962.1705263157895	8.044487278916664	213	1553	991.5	466
SAMN14308592	*T. eigenmanni*	954	757,352	793.8700209643606	7.090557346738465	221	2276	810	144
SAMN14308593	*T. eigenmanni*	889	429,126	482.7064116985377	5.1267901069138055	150	1249	473	2
SAMN14308594	*T. eigenmanni*	932	582,364	624.8540772532189	6.4647753904877705	208	1143	618.5	36
SAMN14308595	*T. eigenmanni*	918	887,446	966.7167755991286	8.505270843790518	216	1775	985.5	437
SAMN14308596	*T. eigenmanni*	942	898,822	954.1634819532909	9.06908511	218	4119	976	439
SAMN14308597	*T. eigenmanni*	931	900,369	967.0988184747583	7.670443180696365	234	1688	992	458
SAMN14308600	*T. styx*	945	957,683	1013.421164021164	9.422176527747853	172	5368	1054	550
SAMN14308602	*T. subterraneus*	952	1,168,756	1227.68487	10.132327221992686	263	2674	1233	741
SAMN14308603	*T. subterraneus*	956	972,331	1017.0826359832636	8.09591782	234	2019	1061	571
SAMN14308604	*T. subterraneus*	953	913,042	958.0713536201469	8.144473985036392	218	1585	990	459
SAMN14308605	*T. styx*	917	989,867	1079.4623773173391	10.216458737931132	196	1892	1129	606
SAMN14308606	*T. styx*	949	1,106,360	1165.8166491043203	10.13116374710511	261	2749	1175	696
SAMN14308607	*T. styx*	926	1,089,755	1176.841252699784	10.836513063195257	228	1981	1228	688
SAMN14308608	*T. styx*	943	1,028,402	1090.5641569459174	9.116641132964514	156	1645	1139	649
SAMN14308609	*T. styx*	904	554,610	613.5066371681415	6.914857689265122	182	1938	613.5	21
SAMN14308610	*T. styx*	968	946,943	978.2469008264463	8.07287576	199	1681	1020	519
SAMN14308611	*T. styx*	934	1,044,744	1118.5695931477517	8.45502434	203	1743	1149	662
SAMN14308612	*T. styx*	970	740,599	763.5041237113402	6.4561363027596474	86	1408	778.5	99
SAMN14308613	*T. styx*	954	965,448	1012	7.934619440772229	191	1678	1036.5	541
SAMN14308614	*T. styx*	935	1,045,700	1118.3957219251338	9.509232879073718	212	4282	1150	652
SAMN14308615	*T. styx*	944	952,634	1009.146186440678	8.103944415840092	216	2832	1033	530
SAMN14308616	*T. styx*	987	612,286	620.3505572441743	5.429247045162959	207	1119	627	11
SAMN14308617	*T. subterraneus*	954	933,975	979.0094339622641	8.548892669331659	211	4150	997	473
SAMN14308618	*T. styx*	940	1,178,711	1253.9478723404254	10.18565190060161	173	2256	1278	753
SAMN14308620	*T. styx*	958	802,060	837.223382	6.997163663906387	211	1800	846.5	208
SAMN14308621	*T. styx*	944	634,440	672.0762711864406	6.538771097749538	56	1436	681	47
SAMN14308622	*T. styx*	972	621,747	639.6574074074074	6.09939225	209	1167	643	25
SAMN14308623	*T. styx*	948	647,064	682.5569620253165	6.449984451657744	211	1280	684	48
SAMN14308624	*T. subterraneus*	939	1,010,552	1076.2002129925452	9.919792415337152	226	2381	1119	598
SAMN14308625	*T. subterraneus*	920	818,322	889.4804347826087	9.283765767037828	162	2586	899	344
SAMN14308626	*T. subterraneus*	962	624,359	649.0218295218295	5.743966893322675	215	1177	660	18
SAMN14308627	*T. styx*	937	1,142,618	1219.4429028815368	10.019530340131116	284	2281	1249	729
SAMN14308628	*T. styx*	942	1,128,838	1198.3418259023354	9.650236100112656	258	2079	1220	728
SAMN14308629	*T. styx*	946	593,493	627.3710359408034	5.989393211481486	208	1157	633.5	15
SAMN14308630	*T. styx*	966	601,776	622.9565217391304	5.824504109818181	210	1145	626.5	12
SAMN14308631	*T. styx*	969	642,120	662.6625386996905	6.046650304075834	208	1192	676	32
SAMN14308632	*T. styx*	976	666,788	683.1844262295082	6.286604519808859	207	2289	689	36
SAMN14308633	*T. styx*	956	618,121	646.5700836820083	6.367672259101868	193	1819	651	32
SAMN14308634	*T. styx*	923	1,267,163	1372.8743228602384	11.751984944551182	212	2670	1394	781
SAMN14308635	*T. styx*	963	852,646	885.4060228452752	7.308677720200425	208	1661	901	323
SAMN14308637	*T. subterraneus*	916	908,970	992.3253275109171	9.715658319191547	213	2434	1009	472
SAMN14308638	*T. styx*	918	1,144,229	1246.4368191721132	11.100842353365447	251	2384	1262	704
SAMN14308639	*T. styx*	952	950,456	998.3781512605042	8.481539017547982	208	1702	1035	532
SAMN14308640	*T. styx*	956	980,699	1025.8357740585775	10.61518621499756	223	2368	1039.5	518
SAMN14308641	*T. styx*	980	607,347	619.7418367346938	5.463554716461212	210	1124	625.5	7
SAMN14308642	*T. subterraneus*	946	887,943	938.6289640591966	8.294843881023548	219	1751	956	394
SAMN14308643	*T. subterraneus*	964	600,873	623.3122406639004	6.0071884344997715	209	1365	622.5	23
SAMN14308644	*T. subterraneus*	947	657,629	694.4340021119324	6.164807711833772	187	1653	700	43
SAMN14308645	*T. subterraneus*	965	624,874	647.5378238341968	6.41028307	208	1252	648	34
SAMN14308646	*T. styx*	951	589,686	620.0694006309149	6.255056412266459	208	1115	626	25
SAMN14308647	*T. subterraneus*	954	612,167	641.6844863731657	6.087699395902728	211	1224	647	27
SAMN14308648	*T. styx*	947	571,307	603.2808870116156	5.94427672	208	1776	602	9
SAMN14308649	*T. styx*	962	839,691	872.8596673596674	8.292276229792325	167	1728	911.5	326
SAMN14308650	*T. styx*	948	979,115	1032.821729957806	9.517736822812637	209	1654	1090	585
SAMN14308651	*T. subterraneus*	950	848,671	893.3378947368421	7.853985022525661	186	1604	917	337
SAMN14308652	*T. styx*	946	1,045,517	1105.1976744186047	9.65954601	212	2556	1109	617
SAMN14308654	*T. styx*	960	900,841	938.3760416666667	7.930145390216616	223	1878	951	409
SAMN14308655	*T. styx*	921	1,074,511	1166.6786102062974	10.917265288616436	199	2048	1223	662
SAMN14308656	*T. styx*	945	964,259	1020.3798941798942	9.2088127	199	1726	1061	555
SAMN14308657	*T. styx*	940	932,866	992.4106382978723	10.086965419863654	221	1663	1041.5	527
SAMN14308658	*T. styx*	925	1,044,875	1129.5945945945946	11.990296034109306	162	3927	1164	619
SAMN14308659	*T. styx*	940	997,134	1060.7808510638297	10.681777912439204	217	2015	1113	583
SAMN14308660	*T. styx*	940	544,719	579.4882978723405	5.938155587838043	210	1164	587	7
SAMN14308661	*T. styx*	932	548,443	588.4581545064377	6.237779722859307	212	1255	583	18
SAMN14308662	*T. styx*	926	536,993	579.9060475161987	5.844388288663213	207	1328	577	9
SAMN14308663	*T. styx*	958	717,363	748.8131524008351	7.099809278228976	207	1343	759.5	121
SAMN14308664	*T. styx*	953	584,731	613.5687303252886	6.5970026365554215	132	1268	618	26
SAMN14308665	*T. styx*	918	443,710	483.3442265795207	5.372611663590224	212	995	464	0
SAMN14308666	*T. subterraneus*	961	933,726	971.6191467221644	9.319726967694027	224	2001	985	453
SAMN14308667	*T. styx*	927	528,964	570.6192017259979	6.465833654767023	165	1178	574	15
SAMN14308668	*T. styx*	931	531,771	571.1825993555317	6.268838184842421	208	1600	571	15
SAMN14308669	*T. subterraneus*	869	340,406	391.72151898734177	3.9386644905688257	188	821	376	0
SAMN14308670	*T. subterraneus*	963	879,976	913.7860851505711	7.807290073594988	208	1905	939	377
SAMN14308671	*T. styx*	966	928,772	961.4616977225672	7.947965415247053	232	2457	959	406
SAMN14308672	*T. styx*	962	932,002	968.8170478170479	8.310600721911861	184	1809	974	445
SAMN14308673	*T. styx*	948	991,965	1046.376582278481	8.924517319608523	212	1877	1095	594
SAMN14308674	*T. styx*	928	921,193	992.6648706896551	8.699388969217857	209	2181	1024.5	502
SAMN14308675	*T. styx*	960	1,005,514	1047.4104166666666	8.55266597	199	2008	1057	581

**Table 4:** Biosample IDs for sequences used in phylogenetic analyses of mitochondrial DNA.

Genbank ID	Species	Specimen Code	Locality	Drainage	County	State
HQ707798	*T. styx*	MLN MKN1	McKinney Pit	Tennessee	Colbert	AL
HQ707784	*T. styx*	MLN MKN3	McKinney Pit	Tennessee	Colbert	AL
JN592319	*T. styx*	Tsub14841	McKinney Pit	Tennessee	Colbert	AL
JN592318	*T. styx*	TsubMKN2	McKinney Pit	Tennessee	Colbert	AL
HQ707780	*T. styx*	MLN GCR1	Guess Creek Cave	Tennessee	Jackson	AL
HQ707801	*T. styx*	MLN SAR6	Salt River Cave	Tennessee	Jackson	AL
JN592361	*T. styx*	TsubSAR1	Salt River Cave	Tennessee	Jackson	AL
JN592362	*T. styx*	TsubSAR2	Salt River Cave	Tennessee	Jackson	AL
JN592363	*T. styx*	TsubSAR3	Salt River Cave	Tennessee	Jackson	AL
JN592364	*T. styx*	TsubSAR4	Salt River Cave	Tennessee	Jackson	AL
JN592320	*T. styx*	TsubDAN311	Davis Bat Cave	Tennessee	Lauderdale	AL
JN592326	*T. styx*	TsubKYC01	Key Cave	Tennessee	Lauderdale	AL
JN592327	*T. styx*	TsubKYC02	Key Cave	Tennessee	Lauderdale	AL
HQ707781	*T. styx*	MLN WSP1	White Spring Cave	Tennessee	Limestone	AL
JN592321	*T. styx*	TsubDAN288	Bobcat Cave	Tennessee	Madison	AL
HQ707794	*T. styx*	UAIC 14,148.01	Muddy Cave	Tennessee	Madison	AL
HQ707796	*T. styx*	MLN SHL2	Shelta Cave	Tennessee	Madison	AL
JN592322	*T. styx*	TsubSHL1	Shelta Cave	Tennessee	Madison	AL
JN592323	*T. styx*	TsubSHL3	Shelta Cave	Tennessee	Madison	AL
JN592324	*T. styx*	Tsub14821	Beech Spring Cave	Tennessee	Marshall	AL
JN592325	*T. styx*	Tsub14842	Cave Spring Cave	Tennessee	Morgan	AL
JN592328	*T. eigenmanni*	TsubNFL01	Norfolk Lake	White	Baxter	AR
HQ707770	*T. eigenmanni*	MLN AR27	Alexander Cave	White	Stone	AR
HQ707792	*T. eigenmanni*	MLN AR28	Alexander Cave	White	Stone	AR
HQ707790	*T. eigenmanni*	MLN AR29	Ennis Cave	White	Stone	AR
HQ707795	*T. styx*	DAN 287	Limestone Caverns	Tennessee	Dade	GA
JN592329	*T. styx*	TsubGEN99	Limestone Caverns	Tennessee	Dade	GA
HQ707785	*T. styx*	MLN LRW1	Longs Rock Wall Cave	Tennessee	Dade	GA
HQ707817	*T. styx*	MLN LRW3	Longs Rock Wall Cave	Tennessee	Dade	GA
JN592330	*T. styx*	TsubLRW2	Longs Rock Wall Cave	Tennessee	Dade	GA
JN592392	*T. subterraneus*	TsubLN1	L & N Railroad Cave	Green	Barren	KY
JN592393	*T. subterraneus*	TsubLN2	L & N Railroad Cave	Green	Barren	KY
JN592394	*T. subterraneus*	TsubLN3	L & N Railroad Cave	Green	Barren	KY
JN592395	*T. subterraneus*	TsubLN4	L & N Railroad Cave	Green	Barren	KY
JN592396	*T. subterraneus*	TsubM1	Mammoth Cave	Green	Edmonson	KY
JN592397	*T. subterraneus*	TsubM2	Mammoth Cave	Green	Edmonson	KY
JN592398	*T. subterraneus*	TsubM3	Mammoth Cave	Green	Edmonson	KY
JN592399	*T. subterraneus*	TsubM4	Mammoth Cave	Green	Edmonson	KY
JN592400	*T. subterraneus*	TsubS1	Sanders Cave	Green	Edmonson	KY
JN592401	*T. subterraneus*	TsubS2	Sanders Cave	Green	Edmonson	KY
JN592402	*T. subterraneus*	TsubS3	Sanders Cave	Green	Edmonson	KY
JN592403	*T. subterraneus*	TsubS4	Sanders Cave	Green	Edmonson	KY
HQ707816	*T. subterraneus*	MLN 0235	Daves Cave	Cumberland	Pulaski	KY
HQ707815	*T. subterraneus*	MLN 0236	Daves Cave	Cumberland	Pulaski	KY
JN592334	*T. subterraneus*	TsubMLN240	Daves Cave	Cumberland	Pulaski	KY

**Table 4:** Continued

Genbank ID	Species	Specimen Code	Locality	Drainage	County	State
JN592333	*T. subterraneus*	TsubMLN475	Drowned Rat Cave	Cumberland	Pulaski	KY
JN592332	*T. subterraneus*	TsubMLN530	Drowned Rat Cave	Cumberland	Pulaski	KY
JN592331	*T. subterraneus*	TsubSA2	Drowned Rat Cave	Cumberland	Pulaski	KY
HQ707788	*T. subterraneus*	MLN 0454	Wells Cave	Cumberland	Pulaski	KY
HQ707809	*T. eigenmanni*	MLN 0414	Carroll Cave	Osage	Camden	MO
HQ707787	*T. eigenmanni*	MLN 0427	Carroll Cave	Osage	Camden	MO
HQ707808	*T. eigenmanni*	MLN 0431	Carroll Cave	Osage	Camden	MO
JN592335	*T. eigenmanni*	TsubMLN439	Carroll Cave	Osage	Camden	MO
JN592337	*T. eigenmanni*	TsubMLN347	Coalbank Cave	White	Carter	MO
JN592336	*T. eigenmanni*	TsubMLN482	Coalbank Cave	White	Carter	MO
JN592338	*T. eigenmanni*	TsubMLN494	Coalbank Cave	White	Carter	MO
JN592339	*T. eigenmanni*	TsubCON3	Concolor Cave	White	Howell	MO
JN592340	*T. eigenmanni*	TsubMLN468	Concolor Cave	White	Howell	MO
JN592341	*T. eigenmanni*	TsubMLN487	Concolor Cave	White	Howell	MO
JN592342	*T. eigenmanni*	TsubBC5	Bliss Camp Cave	White	Oregon	MO
JN592343	*T. eigenmanni*	TsubMLN394	Bliss Camp Cave	White	Oregon	MO
HQ707810	*T. eigenmanni*	MLN 0399	Falling Spring Cave	White	Oregon	MO
HQ707811	*T. eigenmanni*	MLN 0429	Falling Spring Cave	White	Oregon	MO
HQ707807	*T. eigenmanni*	MLN 0391	Posy Spring Cave	White	Oregon	MO
HQ707806	*T. eigenmanni*	MLN 0426	Posy Spring Cave	White	Oregon	MO
HQ707814	*T. eigenmanni*	MLN PS2	Posy Spring Cave	White	Oregon	MO
JN592344	*T. eigenmanni*	TsubMLN495	Posy Spring Cave	White	Oregon	MO
HQ707812	*T. eigenmanni*	MLN 0357	Roaring Spring Cave	White	Oregon	MO
HQ707813	*T. eigenmanni*	MLN RS4	Roaring Spring Cave	White	Oregon	MO
JN592345	*T. eigenmanni*	TsubMLN435	Roaring Spring Cave	White	Oregon	MO
JN592346	*T. eigenmanni*	TsubTS6	Turner Spring Cave	White	Oregon	MO
JN592348	*T. eigenmanni*	TsubMLN469	Panther Cave	White	Ripley	MO
JN592347	*T. eigenmanni*	TsubRIP002	Panther Cave	White	Ripley	MO
JN592349	*T. eigenmanni*	TsubMLN422	Brawley Cave	White	Shannon	MO
JN592350	*T. eigenmanni*	TsubMLN404	Flying W Cave	White	Shannon	MO
JN592351	*T. eigenmanni*	TsubMLN433	Flying W Cave	White	Shannon	MO
HQ707805	*T. styx*	MLN BS5	Blowing Springs Cave	Tennessee	Coffee	TN
JN592352	*T. styx*	TsubBS2	Blowing Springs Cave	Tennessee	Coffee	TN
JN592353	*T. styx*	TsubBS3	Blowing Springs Cave	Tennessee	Coffee	TN
JN592354	*T. styx*	TsubBS4	Blowing Springs Cave	Tennessee	Coffee	TN
JN592355	*T. styx*	TsubMLN296	Baugus Cave	Tennessee	Decatur	TN
JN592356	*T. styx*	TsubMLN305	Baugus Cave	Tennessee	Decatur	TN
JN592357	*T. styx*	TsubMLN306	Baugus Cave	Tennessee	Decatur	TN
HQ707783	*T. styx*	UTTC 04,809	Baugus Cave	Tennessee	Decatur	TN
HQ707804	*T. styx*	MLN GAR4	Garner Spring Cave	Tennessee	Franklin	TN
JN592358	*T. styx*	TsubGAR1	Garner Spring Cave	Tennessee	Franklin	TN
JN592359	*T. styx*	TsubGAR2	Garner Spring Cave	Tennessee	Franklin	TN
JN592360	*T. styx*	TsubGAR3	Garner Spring Cave	Tennessee	Franklin	TN
JN592388	*T. styx*	TsubLCCC01	Little Crow Creek Cave	Tennessee	Franklin	TN
JN592389	*T. styx*	TsubLCCC02	Little Crow Creek Cave	Tennessee	Franklin	TN
JN592367	*T. styx*	Tsub4814	Big Mouth Cave	Tennessee	Grundy	TN
JN592365	*T. styx*	TsubBM8	Big Mouth Cave	Tennessee	Grundy	TN
JN592366	*T. styx*	TsubBM9	Big Mouth Cave	Tennessee	Grundy	TN
HQ707777	*T. styx*	UTTC 04,821	Big Mouth Cave	Tennessee	Grundy	TN
JN592368	*T. styx*	Tsub4812	Crystal Cave	Tennessee	Grundy	TN
JN592369	*T. styx*	Tsub4813	Crystal Cave	Tennessee	Grundy	TN
HQ707776	*T. styx*	UTTC 04,811	Crystal Cave	Tennessee	Grundy	TN
HQ707789	*T. styx*	UTTC 04,820	Trussell Cave	Tennessee	Grundy	TN
HQ707782	*T. styx*	UTTC 04,823	Cave Branch Cave	Tennessee	Hickman	TN
HQ707802	*T. styx*	UTTC 04,822	Allens Creek Cave	Tennessee	Lewis	TN
HQ707786	*T. styx*	MLN LPC1	Lost Pig Cave	Tennessee	Marion	TN
JN592370	*T. styx*	TsubPSC01	Pryor Cave Spring	Tennessee	Marion	TN
JN592371	*T. styx*	Tsub4807	Gallagher Cave South	Tennessee	Marshall	TN
HQ707793	*T. styx*	UTTC 04,808	Gallagher Cave South	Tennessee	Marshall	TN
HQ707774	*T. styx*	UTTC 04,810	Pompie Cave	Tennessee	Maury	TN
HQ707803	*T. styx*	UTTC 04,806	East Water Supply Cave	Cumberland	Overton	TN
HQ707771	*T. styx*	MLN 0026	Anderson Spring Cave	Cumberland	Putnam	TN
JN592372	*T. styx*	TsubMLN027	Anderson Spring Cave	Cumberland	Putnam	TN
JN592373	*T. styx*	Tsub4825	Bartlett Cave	Cumberland	Putnam	TN
HQ707800	*T. styx*	UTTC 04,824	Bartlett Cave	Cumberland	Putnam	TN
JN592390	*T. styx*	TsubBFC01	Blind Fish Cave	Cumberland	Putnam	TN
JN592391	*T. styx*	TsubBFC02	Blind Fish Cave	Cumberland	Putnam	TN
JN592374	*T. styx*	TsubMLN001	Jacques Cave	Cumberland	Putnam	TN
JN592375	*T. styx*	TsubMLN002	Jacques Cave	Cumberland	Putnam	TN
JN592376	*T. styx*	TsubMLN003	Jacques Cave	Cumberland	Putnam	TN
JN592378	*T. styx*	TsubMLN289	Stamps Cave	Cumberland	Putnam	TN
JN592377	*T. styx*	TsubMLN337	Stamps Cave	Cumberland	Putnam	TN
HQ707778	*T. styx*	MLN 0259	Sinking Ridge Cave	Cumberland	Robertson	TN
HQ707791	*T. styx*	MLN JAC1	Sinking Ridge Cave	Cumberland	Robertson	TN
JN592379	*T. styx*	Tsub4818	Herring Cave	Cumberland	Rutherford	TN
HQ707779	*T. styx*	UTTC 04,817	Herring Cave	Cumberland	Rutherford	TN
HQ707797	*T. styx*	UTTC 04,819	Herring Cave	Cumberland	Rutherford	TN
JN592380	*T. styx*	TsubPT2	Pattons Cave	Cumberland	Rutherford	TN
JN592381	*T. styx*	TsubPT5	Pattons Cave	Cumberland	Rutherford	TN
JN592382	*T. styx*	TsubPT6	Pattons Cave	Cumberland	Rutherford	TN
JN592383	*T. styx*	TsubPT7	Pattons Cave	Cumberland	Rutherford	TN
HQ707775	*T. styx*	MLN FLR2	Flat Rock Cave	Cumberland	Smith	TN
JN592384	*T. styx*	TsubFLR1	Flat Rock Cave	Cumberland	Smith	TN
HQ707769	*T. styx*	YFTC1453	Camps Gulf Cave	Cumberland	Van Buren	TN
JN592385	*T. styx*	Tsub4816	Camps Gulf Cave No. 2	Cumberland	Van Buren	TN
HQ707799	*T. styx*	UTTC 04,815	Camps Gulf Cave No. 2	Cumberland	Van Buren	TN
HQ707772	*T. styx*	MLN BLW1	Blowing Cave	Cumberland	Warren	TN
HQ707773	*T. styx*	MLN JS3	Jaco Spring Cave	Cumberland	Warren	TN
JN592387	*T. styx*	TsubJS10	Jaco Spring Cave	Cumberland	Warren	TN
JN592386	*T. styx*	TsubJS2	Jaco Spring Cave	Cumberland	Warren	TN

### Specimen sampling and sequence assembly

We analyzed both the *ND2* mitochondrial DNA locus and ultraconserved elements (UCEs), a type of genomic marker (Faircloth et al. 2012), to elucidate the relationships of major lineages of *Typhlichthys* cavefishes. We assembled a composite ultraconserved element dataset by combining previously published UCE data for cavefishes (Hart et al. 2020; Ghezelayagh et al. 2022; Hart et al. 2024) with UCEs mined from whole genomes using *phyluce* v. 1.7.3 (Faircloth 2016) with the acanthomorph-specific UCE probe set (Alfaro et al. 2018). After filtering using *phyluce* v. 1.7.3 to remove duplicate UCEs, using *CIAlign* (Tumescheit et al. 2022) to visualize and remove chimeric sequences, and using MAFFT via *phyluce* v. 1.7.3 to align sequences (Katoh and Toh 2008; Katoh and Standley 2013), we retained 920 UCEs for 83 *Typhlichthys* specimens and 10 outgroups, including all genera in *Percopsiformes* and its sister taxon *Polymixia* (Ghezelayagh et al. 2022; Near and Thacker 2024). For the *ND2* alignment, we downloaded all 145 sequences available for *Typhlichthys* from the NCBI Genbank Repository and used two specimens of *Speoplatyrhinus poulsoni*, four specimens of *Troglichthys rosae*, and three specimens of *Chologaster cornuta* as outgroups. Both the mtDNA and UCE datasets sample individuals from all major lineages of *Typhlichthys*, including all three species as delimited here.

### Phylogenetic analyses

We conducted phylogenetic analyses of the UCE and mtDNA sequence alignments under maximum likelihood using *IQ-TREE2* (Nguyen et al. 2015; Minh, Schmidt, et al. 2020). We ran *IQ-TREE2* separately for each UCE sequence alignment to produce gene trees and also ran analyses alternatively treating the concatenated UCEs as one or several partitions. For each gene tree and the two analyses of the concatenated sequences, we selected best-fit models of molecular evolution using *ModelFinder* (Kalyaanamoorthy et al. 2017) and estimated nodal support using ultrafast bootstrap values. Using the gene trees, we inferred a species tree under the multispecies coalescent implemented in *ASTRAL*-III (Zhang et al. 2018). We also calculated gene and site concordance factors, which measure the proportion of decisive gene trees and sites that are consistent with resolution of a particular node in an input species tree, using *IQ-TREE2* (Minh, Hahn, et al. 2020). For this analysis, we used the single partition concatenated phylogeny as the input phylogeny. We used the IQ-TREE web server (Trifinopoulos et al. 2016) to analyze the mtDNA alignment and infer a tree with ultrafast bootstrap and SH-like approximate likelihood ratio test (SH-aLRT) (Guindon et al. 2010) supports for each node. Finally, we ran a Bayesian phylogenetic analysis of the mtDNA alignment using *MrBayes* v 3.2.7 (Huelsenbeck and Ronquist 2001; Ronquist et al. 2012) where we partitioned sequences by codon position and used an HKY + I + G model. We ran the Bayesian analysis over 4 chains for 10 million generations (sampling every thousand), checked for convergence of the posteriors and effective sample size values over 200 using Tracer v 1.7.1 (Rambaut et al. 2018), and summarized the posterior trees in a single maximum clade credibility tree.

### Species delimitation and population genetics

We analyzed both the mtDNA sequence alignment and UCE alignment to interrogate population structure and lineage divergence in *Typhlichthys.* First, we calculated pairwise sequence dissimilarity values for all sequences in the alignment using the R package *adegenet* (Jombart 2008). Next, we inferred a haplotype map using the R package *pegas* (Paradis 2010). Finally, we used *Bayesian Phylogenetics and Phylogeography* (*BPP*) v. 4.8.0 (Flouri et al. 2018) to infer input divergence time (τ) and population size (θ) values for calculation of the genealogical divergence index (Jackson et al. 2017) using the equation *gdi* = 1–e^−2τ/θ^ for a subsample of 100% complete matrix UCE alignments containing between 9 and 12 representative individuals (depending on the analytical iteration) for three schemes: one where *Typhlichthys styx* is treated as a single species, one in which *T. styx* is split into southeastern and northern lineages following the phylogenies generated from the UCE loci, and finally one in which *T. styx* is split into its four major constituent lineages following the phylogenies generated from the UCE loci. Two to three individuals were included in each analysis after being selected from our larger UCE sequence dataset based on contig count and completeness [as determined using *phyluce* 1.7.3 (Faircloth 2016); Table 3], as early runs showed the effect of uneven individual sampling per input lineage on *gdi* calculation. For each of these iterations, we fixed an input tree following the topology inferred for the UCE sequences using *IQ-TREE2*, ran each analysis for 1 million generations with a 10,000 generation pre-burnin, and checked for convergence of the posteriors and ESS values over 200 using *Tracer* v. 1.7.1 (Rambaut et al. 2018).

### Divergence time estimation

We inferred time-calibrated phylogenies for *Amblyopsidae* using a node-dating approach in *BEAST* v. 2.6.7 (Bouckaert et al. 2014; Bouckaert et al. 2019). For this analysis, we used the fossil calibrations specified in a recent study (Brownstein et al. 2025) for node dating analysis of *Percopsiformes* and three randomly selected sets of 50 UCE alignments subsampled for representatives of all major *Typhlichthys* lineages, including both the eastern and western lineages of *T. subterraneus* and all four major lineages within *T. styx*. The two fossil calibrations were (1) the root node, which is the *Polymixia-Percopsiformes* split and was set to 97.2 Ma, the age of the oldest Pan-*Polymixia*, with bounds of 66.02 Ma, the age of the oldest *Percopsiformes*, and 125.0 Ma, before which no acanthomorph fossils are known, and (2) the MRCA of *Aphredoderus sayanus* and *Amblyopsidae* (Brownstein et al. 2025). We fixed all internal nodes in the analysis to match the topology of the phylogenies inferred from the UCE alignments under maximum likelihood in *IQ-TREE2* and ran the analysis of each set of UCEs three times independently over 500 million generations with a 100 million generation pre-burnin. Following completion of the MCMC runs, we checked for ESS values over 200 and posterior convergence using *Tracer* v. 1.7.1 (Rambaut et al. 2018), combined all nine posterior tree sets with a 75% burnin and subsampling every 5000 generations using *LogCombiner* v. 2.6.6, and summarized the combined posterior tree set in a maximum clade credibility tree using *TreeAnnotator* v. 2.6.7 (Bouckaert et al. 2019).

### Historical biogeographic reconstruction

We used the R package *BioGeoBEARS* (Matzke 2013) to reconstruct the historical biogeography of *Typhlichthys* across principal aquifers of southeastern North America. *Typhlichthys* represents one of four independent cave colonizations in *Amblyopsidae* (Brownstein et al. 2025), and it would not be logical to reconstruct the historical biogeography of surface-water fishes along subterranean aquifers. Because using the complete time-calibrated phylogeny generated in *BEAST* v. 2.6.7 (Bouckaert et al. 2014; Bouckaert et al. 2019) would necessitate coding all species of amblyopsid for their presence on aquifers and reconstructing ancestral aquifer occupation along nodes representing surface-dwelling common ancestors of amblyopsid genera (Brownstein et al. 2025), we pruned the input time tree to only include lineages of *Typhlichthys.* We coded these lineages as belonging to five principal aquifer systems after plotting their occurrences along aquifer maps (see earlier): Mississippian aquifers, the Ozark Aquifer, Silurian-Devonian aquifers, Valley and Ridge aquifers, and Ordovician aquifers. We conducted ancestral biogeographic reconstructions under three models in *BioGeoBEARS* with and without the + j jump dispersal parameter: a dispersal-extinction-cladogenesis (DEC) model, a dispersal-vicariance-like (DIVALIKE) model, and a Bayesian ancestral biogeography (BAYAREALIKE) model. We selected the best-fitting model by comparing corrected Akaike Information Criterion scores. Using the best-fit model, we ran biogeographic stochastic mapping to estimate the mode and count of biogeographic phenomena (dispersals and vicariant events among areas, founder events, etc.) using simulated biogeographic histories that are congruent with the reconstruction inferred under the input model (Dupin et al. 2017). For the biogeographic stochastic mapping analysis, we inputted 100 as the maximum number of maps to attempt, 50 as the goal number of maps to attempt, and 400 attempts per branch. We extracted counts for founder and sympatry events from the anagenetic event output and plotted them through time.

### Correlation of divergence times with carboniferous aquifer incision

To correlate the timing of fragmentation of aquifers with the divergence times we inferred for major lineages of *Typhlichthys*, we assembled relevant geological data previously used to calibrate the age of the Cumberland River incision, which we hypothesize drove the isolation of the eastern and western Mississippian Aquifer systems. If the incision of these rivers removed pathways for *Typhlichthys* dispersal by eroding through aquifer-holding formations of particular age (for an example in the Cumberland, see Fig. 4 in (Anthony and Granger 2007)), then we expect that the posterior range of divergence times of all species should coincide with or predate the origins of these major rivers along their modern routes. For the age of the incision of the Cumberland River along its modern route, we used the ages of cosmogenic nuclides buried in caves associated with Cumberland drainage by Anthony and Granger (Anthony and Granger 2004). We then plotted these ages against the ages estimated for divergence times between *Typhlichthys subterraneus* western and eastern sublineages and the divergence time between *T. eigenmanni* and *T. subterraneus*.

### High-resolution computed tomography

We used a Nikon XT H 225 ST system at Yale University to visualize the skeletons of individuals of *Typhlichthys styx* and *T. subterraneus* using high-resolution computed tomography. For our comparative dataset, we also used recently published scans of amblyopsids generated by our research group (Brownstein et al. 2025) and used all scans available on Morphosource.org that could be assigned to particular species in *Typhlichthys* to count vertebrae (Table 1). Our CT scan dataset spanned the distribution of *Typhlichthys styx*, allowing us to comprehensively examine the variability in the anatomy of this species. All scans were segmented using 3DSlicer (Kikinis et al. 2014) and rendered using the free software Blender (blender.org). CT scans and their parameters are available on Morphosource.

### Nomenclatural acts

We have registered this published work and its nomenclatural act on ZooBank, the online registration system for the International Code of Zoological Nomenclature (ICZN). Readers can verify the ZooBank Life Science Identifiers (LSIDs) and access associated information by appending the LSIDs to the prefix “http://zoobank.org/.”

The LSID for this publication is: urn: lsid: zoobank.org: pub:34387C2F-0302–494E-B4D8-BDA7AE3F2871.

## Results

### Phylogeography and species diversity of *Typhlichthys* cavefishes

**Fig. 2:**
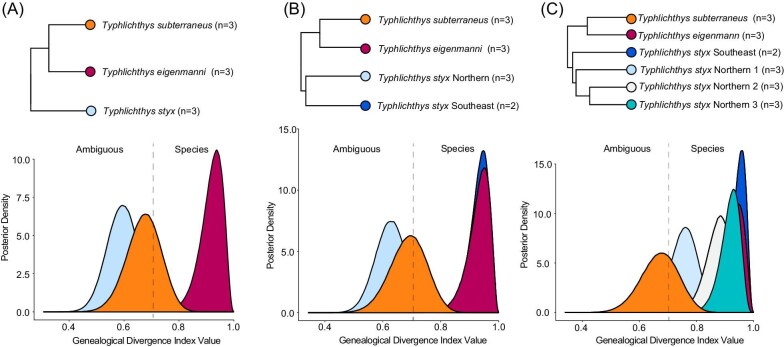
**Recognition of species diversity in *Typhlichthys*.** Plots show posterior densities of genealogical divergence index values for lineages in *Typhlichthys* when *T. styx* is treated as (A) one, (B) two, or (C) four lineages following the recognition of four major clades in phylogenies inferred using ultraconserved elements and mitochondrial data.

Species in the genus *Typhlichthys* are widely distributed across cave ecosystems in the Appalachians across to the Ozarks in eastern North America, but their ranges are not clearly demarcated by major surface river drainages (Fig. 1A; Fig. 2). Instead, *Typhlichthys* are tightly restricted to principal aquifers hosted within rocks of Paleozoic age (Fig. 1B), a pattern that is reflected in phylogenies of the genus inferred using genomic (920 ultraconserved elements; Fig. 1B; Fig. S1-S4) and mitochondrial DNA sequence data (Figs S5-S7); *Typhlichthys eigenmanni* is restricted to carbonate rocks within the Ozark Aquifer in the Ozark Plateau Aquifer System (Niemiller and Poulson 2010), whereas its sister taxon, *T. subterraneus sensu stricto*, is distributed across the aquifers in Mississippian-aged carbonates in the eastern highlands region of Indiana, Kentucky, Tennessee, and Alabama, USA (Fig. 1; Figs S2-S4). The earliest divergence in *T. subterraneus* sensu stricto corresponds to a split between populations occupying the western Mississippian aquifer in Kentucky and Indiana and populations from the eastern Mississippian aquifer in Alabama, Kentucky, and Tennessee, USA (yellow and orange points in Fig. 1).

A third major clade consisting of populations in Tennessee, Alabama, and Georgia, USA, previously considered *T. subterraneus* (Niemiller and Poulson 2010; Niemiller et al. 2012; Niemiller, Graening, et al. 2013; Hart et al. 2024) forms the sister lineage to the clade containing *Typhlichthys eigenmanni* and *T. subterraneus.* The earliest divergence in this lineage, which we name *Typhlichthys styx* sp. nov., corresponds to a split between populations occupying the aquifers within the Valley and Ridge provinces in the southeast and all other populations (Fig. 1B-C)(Niemiller et al. 2012; Niemiller et al. 2016; Hart et al. 2024). The major lineages of *Typhlichthys* all are resolved with high node support in phylogenies inferred using genome-wide markers and mtDNA sequence data (Fig. 1; Figs S2-S7). Species of *Typhlichthys* correspond to three separate haplotype clusters when mapped (Fig. S8), although mitochondrial sequence divergence among lineages within species is also high (Figs S8-S9).

Previous studies have shown using multiple metrics, including the pairwise fixation index (*F_ST_*), that the major lineages of *Typhlichthys* show little to no gene flow (Niemiller et al. 2012; Hart et al. 2024). For example, SNP-based *F_ST_* calculations produce values of 0.463 between *T. eigenmanni* and *T. subterraneus*, and 0.489 to 0.81 between lineages of *T. styx* and these species, suggesting very limited allele sharing (Hart et al. 2024). To further examine patterns of lineage isolation in *Typhlichthys*, we estimated posterior distributions of the genealogical divergence index (*gdi*) estimated using BPP (Leaché et al. 2019) across species and populations of *Typhlichthys* (Fig. 2). The *gdi* was developed to delimit species-level units in a coalescent framework with gene flow without over-splitting lineages as different species (Jackson et al. 2017; Leaché et al. 2019). Posterior distributions of *gdi* for *T. styx* (when treated as one unit) and *T. subterraneus* far exceed the 0.2 threshold for splits among populations (Jackson et al. 2017; Leaché et al. 2019) and match or exceed the *gdi* ranges observed in well-delimited sister species pairs of North American freshwater fishes (Kim et al. 2022; Kim et al. 2023; Ghezelayagh et al. 2025). Although we conservatively do not treat them as distinct species owing to the absence of diagnostic features characterizing each lineage and recent divergence (see later), our results also show that major intraspecific sublineages in *Typhlichthys styx* meet or exceed the *gdi* threshold for species level divergences, which ranges from 0.7 to 1.0 (Jackson et al. 2017; Leaché et al. 2019) (Fig. 2). Although a proportion of the distribution of *gdi* values for *T. subterraneus sensu stricto* and *T. styx* falls within the ambiguous zone of between 0.2 and 0.7, we note that a proportion of *gdi* distributions for species, including other North American fishes (Kim et al. 2022; Ghezelayagh et al.) can commonly fall within this ambiguous zone (Leaché et al. 2019; Opatova et al. 2023; Sporre et al. 2024).

**Fig. 3:**
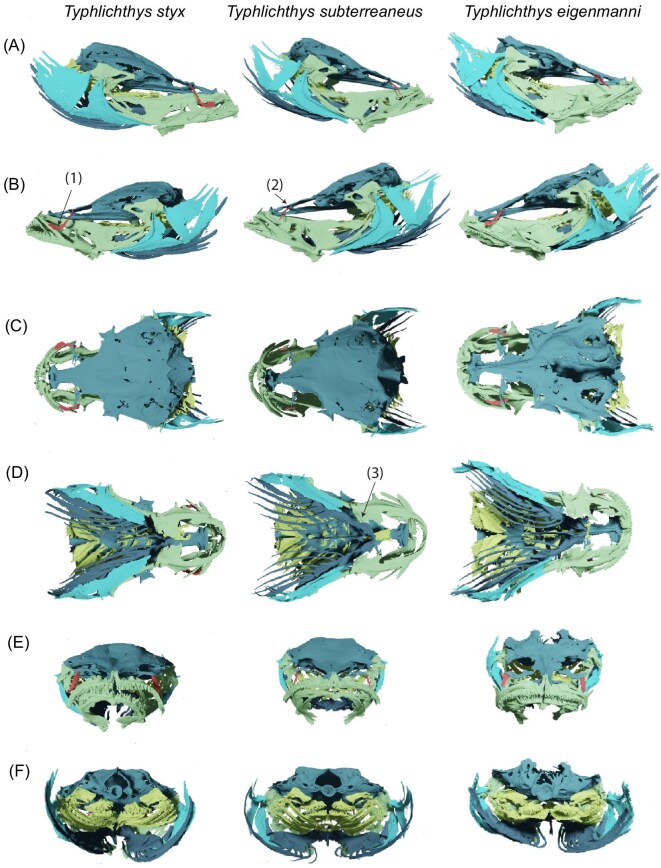
H**igh-resolution computed tomography reveals a new species of *Typhlichthys.*** Rendered skulls of typical representatives of three species of *Typhlichthys* in (A) right lateral, (B) left lateral, (C) dorsal, (D) ventral, (E) anterior, and (F) posterior views showing key features distinguishing species: (1) anteriormost circumorbital (lacrimal) contacts maxilla in *T. styx*, (2) lacrimal is reduced to a thin splint of bone that very briefly contacts lateral ethmoid ventrally in *T. subterranneus*, and (3) retroarticular is not hooked anteriorly in *T. subterraneus* (also see Figure S10). Colors of bone groups in scans indicate: suspensorium (green), circumorbital series (red), neurocranium (aquamarine), opercular series (light blue), branchiostegal series (dark blue), pharyngeobranchial series (yellow). Specimens figured are YPM ICH 25653 (*T. styx*), 25,338 (*T. subterraneus*), and UMMZ 150421 (*T. eigenmanni*).

Although earlier studies, including an analysis of body shape (Burress et al. 2017), failed to distinguish between different species of *Typhlichthys* using morphology, our examination of newly generated high-resolution computed tomography scans of cavefish skeletons and analysis of the evolutionary history of vision-related gene pseudogenization in *Amblyopsidae* (Brownstein et al. 2025) reveal diagnostic characters for all three species in the genus (Fig. 3). Osteological differences include the manner of reduction of the circumorbital series and contact of these bones with other elements of the skull, as well as the morphology of the retroarticular (Fig. 3; Fig. S10). A previous analysis also showed that populations of *Typhlichthys* that belong to *T. styx* are the only known lineages of amblyopsids with a pseudogenized recoverin *rcv2a* (Brownstein et al. 2025). Recoverins accelerate cone cell response recovery and are associated with low light vision in teleost fishes (Zang et al. 2015). Consequently, the loss of a functional *rcv2a* gene in *T. styx* implies that even the genomic basis of low light vision has been subject to relaxed selection pressures in this species, consistent with the hypothesis that *T. styx* is an old obligate subterranean lineage. We note that species of *Typhlichthys* are very similar in skeletal morphology yet are clearly distinguishable from other obligate cave-dwelling amblyopsid genera, all of which independently colonized caves (Niemiller, Fitzpatrick, et al. 2013; Brownstein et al. 2025).

### Systematics

*Typhlichthys styx* sp. nov.

**Phantom cavefish**

**Zoobank registration LSID.** urn: lsid: zoobank.org: act:6DB7E02D-AC77-4688-951A-AFCCCF2EDA9F.

**Holotype.** YPM ICH 025653, YFTC 26,795, adult, 30.22 mm standard length (SL), Flat Rock Cave, Smith County, Tennessee (site MLN 12–41), 36.235°N, 86.0992°W, collected September 24, 2012, by M.L. Niemiller, D. Sores, and G. Moni.

**Paratypes.** YPM ICH 25653 (YFTC 26,796), YPM ICH 25653, YPM ICH 25653, adults, all from the same locality as the holotype and collected at the same time by Niemiller and party.

**Referred material.** See Tables 1-4 for a list of all referred specimens and tissue collections.

**Etymology.** Styx (Ancient Greek Στύξ), the river that forms one of the borders of the underworld in Ancient Greek mythology.

**Diagnosis and description**. *Typhlichthys styx* differs from all other species of *Typhlichthys* in the following combination of features (asterisks indicate autapomorphies): circumorbital series contacts maxilla*; retroarticular strongly anteriorly hooked to form acute posteromedial angle (shared with *T. eigenmanni*); lowest modal number of pectoral fin rays; *Typhlichthys styx* sp. nov. has modally 29 vertebrae, 11 pectoral fin rays, 8 dorsal fin rays, 8 anal fin rays, and 12 branched caudal rays (Table 1; Tables 5-9). The largest examined specimen is 30.22 mm SL.

**Table 5:** Counts of vertebrae in *Typhlichthys styx* sp. nov., *Typhlichthys subterraneus*, and *Typhlichthys eigenmanni. Abbreviations*: N, number of specimens; SD, standard deviation.

	Number of Vertebrae						
Species	28	29	30	31	N	Mean	SD
*Typhlichthys styx*	5	2	3	1	11	29	1.10
*Typhlichthys subterraneus*	7	1			8	28.13	0.35
Typhlichthys eigenmanni.		1	1	1	3	30	0.82

**Table 6:** Counts of pectoral fin rays in *Typhlichthys styx* sp. nov., *Typhlichthys subterraneus*, and *Typhlichthys eigenmanni. Abbreviations*: N, number of specimens; SD, standard deviation.

	Number of Pectoral Fin Rays				
Species	11	12	N	Mean	SD
*Typhlichthys styx*	4	1	5	11.2	0.40
*Typhlichthys subterraneus*	3	6	9	11.67	0.47
*Typhlichthys eigenmanni*		2	2	12	0

**Table 7:** Counts of dorsal fin rays in *Typhlichthys styx* sp. nov., *Typhlichthys subterraneus*, and *Typhlichthys eigenmanni. Abbreviations*: N, number of specimens; SD, standard deviation.

	Number of Dorsal Fin Rays					
Species	8	9	10	N	Mean	SD
*Typhlichthys styx*	3	2		5	8.4	0.55
*Typhlichthys subterraneus*	1	1		2	8.5	0.70
*Typhlichthys eigenmanni*		1	1	2	9.5	0.70

**Table 8:** Counts of anal fin rays in *Typhlichthys styx* sp. nov., *Typhlichthys subterraneus*, and *Typhlichthys eigenmanni. Abbreviations*: N, number of specimens; SD, standard deviation.

	Number of Anal Fin Rays				
Species	8	9	N	Mean	SD
*Typhlichthys styx*	5	1	6	8.16	0.41
*Typhlichthys subterraneus*	1	1	2	8.5	0.70
*Typhlichthys eigenmanni*		2	2	9	0

**Table 9:** Counts of branched caudal fin rays in *Typhlichthys styx* sp. nov., *Typhlichthys subterraneus*, and *Typhlichthys eigenmanni. Abbreviations*: N, number of specimens; SD, standard deviation.

	Number of Branched Caudal Fin Rays						
Species	11	12	13	14	N	Mean	SD
*Typhlichthys styx*		3			3	12	0
*Typhlichthys subterraneus*		8	2	2	12	12.5	0.80
*Typhlichthys eigenmanni*	1	1			2	11.5	0.70

**Geographic distribution.**  *Typhlichthys styx* is distributed throughout the Valley and Ridge, Cumberland Plateau, Interior Low Plateaus, and Nashville Basin of Tennessee, Alabama, and Georgia, USA. *T. styx* includes four or five major lineages endemic to caves corresponding to the Ordovician, Silurian-Devonian, Mississippian, and Valley and Ridge aquifers of southeastern North America (Fig. 1). A complete list of localities where *T. styx* is found is available in Table 10.

**Table 10:** Localities of individual lineages of *Typhlichthys styx*.

Lineage	Locality	Latitude	Longitude
Southeastern	Cave Spring Sink along Graves Creek	34.082569	−86.532984
Southeastern	Crane Creek Cave	34.891940	−85.216670
Southeastern	Sells Cave	34.274720	−85.910560
Northern 1	Allens Creek Cave	35.437500	−87.596390
Northern 1	Baugus Cave	35.752500	−88.085280
Northern 1	Beech Spring Cave	34.438611	−86.458611
Northern 1	Bobcat Cave	34.655833	−86.711111
Northern 1	Herring Cave	35.938610	−86.307500
Northern 1	McKinney Pit	34.725000	−87.791110
Northern 1	Pattons Cave	36.050000	−86.446670
Northern 1	Salt River Cave	34.988330	−85.975560
Northern 1	Shelta Cave	34.754025	−86.611130
Northern 2	Big Mouth Cave	35.332780	−85.826940
Northern 2	Bobcat Cave	34.655833	−86.711111
Northern 2	Garner Spring Cave	35.028610	−85.907220
Northern 2	Geiger Cave	34.820700	−86.053310
Northern 2	Limrock Blowing Cave	34.708890	−86.181390
Northern 2	Little Crow Creek Cave	35.012500	−85.966670
Northern 2	Pattons Cave	36.050000	−86.446670
Northern 2	Shelta Cave	34.754025	−86.611130
Northern 2	Tally Ditch Cave	34.929440	−85.880830
Northern 2	White’s Spring Cave	34.967405	−86.890255
Northern 2	Well, Cave Spring Branch	34.546111	−86.851111
Northern 3	Beech Spring Cave	34.438611	−86.458611
Northern 3	Blowing Springs Cave	35.358802	−85.894192
Northern 3	Bobcat Cave	34.655833	−86.711111
Northern 3	Crystal Cave	35.272780	−85.853610
Northern 3	Flat Rock Cave	36.235000	−86.099170
Northern 3	Gallagher Cave South	35.518610	−86.746940
Northern 3	Hering Cave	34.624360	−86.401610
Northern 3	Jaco Spring Cave	35.786670	−85.660560
Northern 3	Jacques Cave	36.114170	−85.441390
Northern 3	Trussell Cave	35.258610	−85.870830
Northern 3	Twin Cave	36.170211	−87.103513

**Conservation note.** Many populations of *Typhlichthys styx* face threats from anthropogenic activities reducing and contaminating groundwater (Niemiller, Graening, et al. 2013). Habitat alteration, the construction of dams, water over-exploitation, and agricultural and industrial waste are medium to high threats for all known populations of *T. styx* (Niemiller, Graening, et al. 2013).

### Speciation and dispersal in *Typhlichthys* cavefishes are facilitated by aquifers

Our analyses demonstrate that species of *Typhlichthys* and the primary divergences among populations and species appear to correspond to boundaries among the major aquifers hosted within various Paleozoic-aged carbonate rocks of southeastern North America (Anon 2000). Yet, our phylogenetic analyses also show that many cave localities in the Cumberland Plateau harbor multiple species of *Typhlichthys*, and in some cases (Bobcat Cave, Beech Spring Cave, Patton’s Cave, Shelta Cave) multiple sublineages of *T. styx* (Fig. 1, Figs S1-S6; Table S10).

Across our analyses and previous ones, there is no indication of historical introgression or gene flow among delimited species or lineages within species of *Typhlichthys* despite the overlapping ranges of *T. subterraneus* and *T. styx* (Fig. 1, Figs S1-S6)(Hart et al. 2024). The mtDNA and UCE phylogenies sample individuals from across the range of this genus, yet there is no indication of admixture between lineages of *T. subterraneus* and *T. styx* (Hart et al. 2024). All three species of *Typhlichthys* are also recovered as reciprocally monophyletic with high bootstrap support in phylogenies generated using mtDNA; thus, no clear signature of mitochondrial introgression is present in these fishes. The high *F_ST_*values estimated between species of *Typhlichthys* also indicate very low numbers of migrants per generation (*Nm =* 0.058) between *T. styx* and *T. subterraneus*. Together, the consistent, robustly supported reciprocal monophyly of these species and evidence for limited gene flow among them inferred from both nuclear and mitochondrial DNA sequences implies a long history of genetic isolation and limited introgression.

**Fig. 4:**
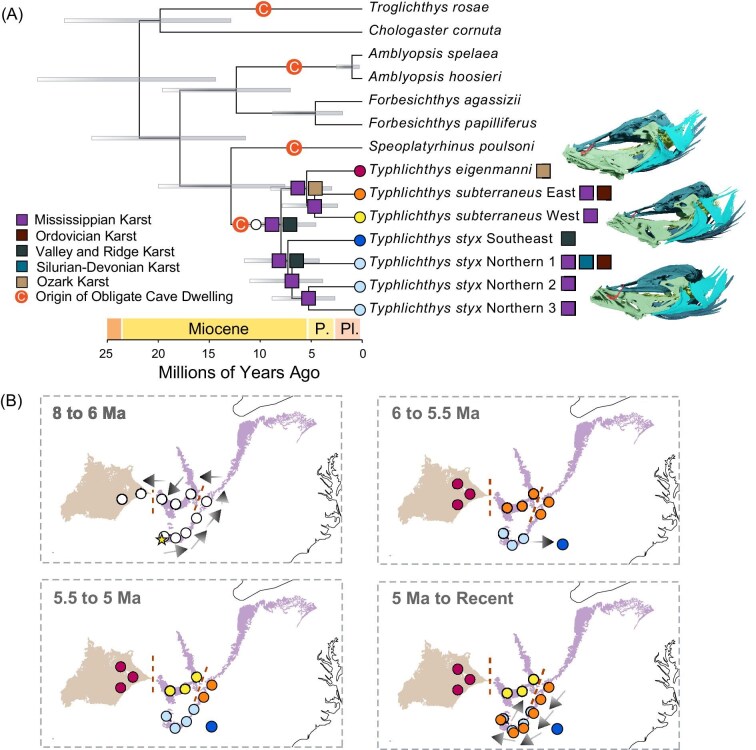
Aquifers **facilitated dispersal and vicariance in *Typhlichthys.***(A) Historical biogeographic reconstruction from the best-fit model in *BioGeoBEARS* (BAYAREALIKE) annotated on the node-dated phylogeny of *Typhlichthys* and other species in *Amblyopsidae* inferred in BEAST 2.6.7. Bars at nodes indicate 95% highest posterior density (HPD) intervals of divergence times. Squares at nodes indicate inferred ancestral areas. Circles at tips indicate species shown on maps in Figure 1 and in Figure 4B. Inferred historical biogeography of *Typhlichthys* is provided in (B). Aquifer colors are the same as in Figure 4 (A) and Figure 1 (B). Arrows indicate direction of key dispersals. Colored circles indicate selected lineages labeled in (A), and yellow star indicates the site of Key Cave, Alabama, USA, where two species of *Typhlichthys* are in sympatry, and the only locality where *Speoplatyrhinus poulsoni*, the sister lineage to *Typhlichthys*, is known to occur. Red dashed lines indicate where connectivity between different aquifers has apparently been reduced, facilitating the allopatric speciation and dispersal of *Typhlichthys*.

Our Bayesian node-dating analysis of the ultraconserved element dataset demonstrates that the most recent common ancestor (MRCA) of the genus *Typhlichthys*, the MRCA of *T. eigenmanni* and *T. subterraneus*, and the common ancestor of intraspecific lineages of *T. styx* are ancient, originating in the Miocene 7.96 Ma (95% highest posterior density, HPD interval: 4.53, 12.73 Ma), 5.44 Ma (95% HPD interval: 2.98, 8.97 Ma), and 7.29 Ma (95% HPD interval: 4.21, 11.57 Ma), respectively (Fig. 4A; Fig. S10). We estimate that intraspecific lineages of *Typhlichthys subterraneus* share most recent common ancestry in the early Pliocene, 4.67 Ma (95% HPD: 2.41, 7.85 Ma). The ancient ages of these successive divergences, which are also supported by the haplotype diversity observed in *T. styx* and *T. subterraneus* (Figs S8-S9), postdate the invasion and obligate cave adaptation of the common ancestor of *Typhlichthys*, which was an obligate cave dweller (Niemiller, Fitzpatrick, et al. 2013; Brownstein et al. 2025; Hart et al. 2025) that had already experienced extensive degeneration of the genomic basis of its vision (Niemiller, Fitzpatrick, et al. 2013; Brownstein et al. 2025).

The ancient divergence times among *Typhlichthys* species, apparent lack of widespread gene flow among species (Hart et al. 2024), and high genealogical divergence (Fig. 2) suggest a long history of reproductive isolation. However, the existence of these lineages in sympatry despite the high levels of aquifer-specific endemicity observed in *Typhlichthys* are contradictory patterns that require explanation. To understand how deeply divergent lineages of *Typhlichthys* are sympatric despite apparently diverging across major aquifer boundaries, we conducted historical biogeographic reconstructions along a time-calibrated phylogeny of *Typhlichthys* using a model-fitting approach Matzke (2013) and treating aquifers as geographic regions. Eastern Mississippian and Ordovician karstic units hold water in solution enlarged openings (Survey 1985), which could conceivably function as subterranean dispersal routes for obligate cave-dwelling freshwater species. Across biogeographic models, our reconstructions show that aquifers within Mississippian-aged karstic carbonate rocks that bracket the Ordovician-rocks exposed in the center of the Nashville Dome structure (Wilson 1935; Stearns and Reesman 1986) have acted as subterranean highways that facilitated dispersals of *Typhlichthys* (Fig. 4).

The favored model (corrected AIC score = 39.42; Table 11) is a simple dispersal-extinction cladogenesis model. Our analyses suggest that the common ancestor of *Typhlichthys* cavefishes resided in cave systems within Mississippian-aged limestones in the Cumberland Plateau province. The subterranean environments within the Mississippian aquifers of the eastern highlands then served as the primary dispersal corridor towards the west and north for both *Typhlichthys styx* and the common ancestor of *T. eigenmanni* and *T. subterraneus* (Fig. 4A; Fig. S12). The earliest divergence in *T. styx* is also along aquifer boundaries; the sister to all other major lineages of *T. styx* is only found in caves associated with the Valley and Ridge aquifers, which we infer represents a dispersal eastward. Our biogeographic reconstruction is also concordant with the observation that *Speoplatyrhinus poulsoni*, which is the sister lineage to *Typhlichthys* (Niemiller and Poulson 2010; Niemiller, Fitzpatrick, et al. 2013; Hart et al. 2020; Brownstein et al. 2025; Hart et al. 2025), probably independently colonized the subterranean environment of Key Cave (the only known locality of *S. poulsoni*), which corresponds to the Mississippian-age Tuscumbia Limestone and Fort Payne Chert in Lauderdale County, Alabama, USA (Ponta et al. 2020). Localities associated with caves within the Mississippian aquifers are also exclusively those where *T. styx* and *T. subterraneus* are found in sympatry (Fig. 1A; Figs S2-S6).

**Table 11:** Comparison of model fit for the BioGeoBears analysis. The best-fit model is bolded.

LnL	Log Likelihood	Corrected AIC score	Corrected AIC score weight
**DEC**	**−16.20806**	**39.41612**	**0.58852700**
DEC + J	−14.44659	42.89318	0.10345027
DIVALIKE	−14.14054	42.28108	0.14049120
DIVALIKE + J	−14.14054	42.28108	0.14049115
BAYAREALIKE	−16.48150	46.96300	0.01352019
BAYAREALIKE + J	−16.48150	46.96300	0.01352019

From these observations and our historical biogeographic construction, we infer a pattern of dispersal in *Typhlichthys* that is concordant with the geographic distribution of aquifers contained by Mississippian-age karstic carbonates. Conceivably, lineages of *Typhlichthys* could have speciated solely via isolation-by-distance (Fig. 1A). However, we also noticed two intriguing phylogeographic patterns in *Typhlichthys* that suggest other mechanisms might modulate speciation in this lineage. First, *Typhlichthys eigenmanni* and *T. subterraneus* are allopatrically distributed on either side of the Mississippi Embayment (Fig. 1). Second, *Typhlichthys styx* and the eastern lineage of *T. subterraneus* exclusively occur south and east of the Cumberland River, and the western lineage of *T. subterraneus* exclusively occurs north of this river (Fig. 1, 4B). River incision inducing the isolation of sister species is inferred to have driven speciation in the cavefish genus *Amblyopsis* in Indiana and Kentucky, USA (Niemiller, McCandless, et al. 2013). Because large river channels tend to be sediment-rich and cloudy, contrasting with the clear, calm pools favored by amblyopsid species (Niemiller, Graening, et al. 2013, et al. 2013; Niemiller et al. 2016), large river channels can act as isolating mechanisms for cave-adapted fishes even when karstic rock is not completely eroded away. However, the divergence times between the eastern and western lineages of *Typhlichthys subterraneus*, as well as between *T. eigenmanni* and *T. subterraneus*, are younger than the Mississippi (Potter-McIntyre et al. 2018) or the Cumberland (Anthony and Granger 2004; Anthony and Granger 2007) drainages. The distribution of divergence times between *Typhlichthys eigenmanni* and *T. subterraneus* also precede the Pleistocene and are therefore unrelated to landscape and drainage changes induced by glaciation. Indeed, lineage divergence has occurred among *Typhlichthys subterraneus* despite the connectivity of Mississippian karst aquifers across the Cumberland drainage in Kentucky (Survey 1985; Noger 1988) at depth and patchily across the landscape (Florea et al. 2002).

Instead, the simplest explanation appears to be that speciation in *Typhlichthys* has occurred via allopatry following rare dispersals in these two points along their distribution (Fig. 4B). River incision and drainage reorganization during this period may have also facilitated the isolation of lineages of *Typhlichthys.* For example, the Cumberland River and its tributaries, which have continuously eroded through Mississippian karstic formations since at least the Miocene (see Fig. 3 in (Anthony and Granger 2007)), may have contributed towards decreased connectivity of eastern and western Mississippian karst since the Miocene. Extensive drainage reorganization also occurred throughout the Tennessee River system during the Pliocene that resulted in its current proximity to the Cumberland Drainage and membership in the larger Mississippi drainage (Odom and Granger 2022); the dispersal of *Typhlichthys styx* across southeastern North America may have been aided by this drainage reorganization event.

Mississippian karst ecosystems are present throughout southern Illinois in the Shawnee Hills region (Panno et al. 2021), which provide a potential bridge between the karst containing the Ozark aquifer in Arkansas, western Illinois, and Missouri, and the karst aquifer systems of the eastern highlands (Fig. 1) that is visible in the phylogeography of other cave-adapted species (Katz et al. 2018; Cucalón et al. 2024). It is notable that only two dispersals, one across the Cumberland and one across the northern rim of the Mississippi Embayment, are needed to explain the distribution of *Typhlichthys* lineages, whereas the widespread sympatry of *T. styx* and *T. subterraneus* in the eastern Mississippian karst aquifer (Fig. 4A) is indicative of continuous dispersal through this geological structure. The evidence available is consistent with long-distance dispersal across these regions, perhaps via surface waters in the regions where aquifers are patchy but present. A dispersal scenario for *Typhlichthys* directly across the Mississippi Embayment is especially unlikely given that Paleozoic rocks that could conceivably produce karst dip up to a kilometer or more underground in the Embayment center. Rare dispersal of *Typhlichthys* across patchy karst landscapes would recall long-distance dispersal and founder event speciation in surface-dwelling lineages, including the arrival of species on islands via rafting (Gillespie et al. 2012).

## Discussion

Subterranean speciation is a poorly understood process that has confounded our ability to reconstruct the evolution of biodiverse cave ecosystems (Poulson and White 1969; Barr and Holsinger 1985; Wiens et al. 2003; Hadid et al. 2013; Li et al. 2015; Culver and Pipan 2019; Li et al. 2020; Langille et al. 2021; Milks et al. 2024). Here, we have shown that lineage divergence has occurred *in situ* in blind, obligate cave-dwelling fishes in the genus *Typhlichthys*, a process that is associated with aquifer boundaries in southeastern North America. Previous work has demonstrated that vision-related genes degenerated before the divergence of species in *Typhlichthys* (Brownstein et al. 2025), providing a rare example of speciation following the acquisition of an obligate subterranean ecology. Although the majority of cave invasions by other amblyopsid cavefishes appear to be independent (Niemiller, Fitzpatrick, et al. 2013, et al. 2013; Brownstein et al. 2025; Hart et al. 2025), our analyses substantiate a case of *in situ* speciation in the subterranean environment.

Our use of ultraconserved elements and mtDNA to infer lineage relationships in *Typhlichthys* and high-resolution computed tomography to examine the osteology of these cavefishes allows us to delimit and describe a new species in this widespread clade. Although the *Typhlichthys* species complex has been highlighted as a source of cryptic subterranean biodiversity threatened by aquifer overuse and mismanagement (Niemiller et al. 2012; Niemiller, Graening, et al. 2013; Hart et al. 2024), the absence of diagnostic differences in body shape, body size, and other morphological characters have inhibited the description of putative new species (Burress et al. 2017; Hart et al. 2020). Our observation that a set of lineages forms a clade sister to *T. subterraneus sensu stricto* and *T. eigenmanni* that is distinguishable on the basis of features of the skull allows us to untangle the Gordian knot of *Typhlichthys* species diversity and describe a third species in this genus.

The phylogenetic relationships and timescale of divergence in species of *Typhlichthys* imply a scenario where speciation was primarily associated with the isolation of Carboniferous-aged aquifers surrounding the southern Appalachians and Ozarks that previously served as dispersal routes. The central importance of Mississippian karst-contained aquifers as the primary route of dispersal is underscored by the coexistence of sympatric species of *Typhlichthys* throughout caves associated with Mississippian aquifer systems in Tennessee and Alabama (Fig. 1), and illuminates a history of subterranean allopatric speciation and secondary sympatry without apparent widespread hybridization (Niemiller et al. 2012; Hart et al. 2020; Hart et al. 2024) among deeply divergent lineages of cavefishes sharing common ancestry in the Miocene (Fig. 4).

Reconciling these biogeographic patterns allows us to reconstruct an evolutionary scenario for *Typhlichthys*, including the deeply divergent sister lineage to the clade containing *T. subterraneus* and *T. eigenmanni*, which we describe as a new species, *Typhlichthys styx*. The ancient ages of intraspecific lineages within *T. styx* and *T. subterraneus* estimated in our node-dated phylogeny (Fig. 4) are consistent with the relative divergence times estimated using the program BPP (Bayesian Phylogenetics and Phylogeography; Supplementary Information) and the substantial mtDNA haplotype diversity observed in both of these species. The absence of any clear diagnostic phenotypic features or mutations of interest among lineages contained within these species leads us to consider them both to be very long-lived species harboring significant (Fig. 2) genetic diversity. In the extensive karstic cave ecosystems of southeastern North America, the same processes have probably contributed to speciation across the Tree of Life, resulting in the formation of regionally endemic faunas (Christman et al. 2005; Niemiller and Zigler 2013) and fueling the origination of ancient clades with a high proportion of subterranean diversity that are endemic to the American southeast, including cave-adapted cholevine (Leray et al. 2019), trechine (Benito et al. 2025), and platynine (Gómez et al. 2016) beetles, cambarid crayfishes (Buhay et al. 2007; Stern et al. 2017), *Tetracion* millipedes (Loria et al. 2011), tomocerid springtails (Katz et al. 2018), phalangodid harvestmen (Hedin and Thomas 2010), *Nesticus* spiders (Hedin 1997; Snowman et al. 2010), asellid isopods (Lewis et al. 2023; Saclier et al. 2024; Tomoyasu et al. 2025), and plethodontid salamanders (Niemiller et al. 2008). Together, these results provide a new set of expectations for delimiting subterranean species and identifying the drivers of their diversity. In an era where groundwater resources are ever more strained due to a combination of resource overuse and aquifer degradation (Aeschbach-Hertig and Gleeson 2012; Famiglietti 2014; Devitt et al. 2019; Jurgens et al. 2022; Jasechko et al. 2024; Saccò et al. 2024), careful reconstruction of the phylogeny of morphologically conservative, widespread lineages will be essential for describing species from underground.

## Supplementary Material

obag021_Supplemental_File

## Data Availability

All CT scans used for this study are on Morphosource.org. New CT scans, as well as previously deposited data, are listed in Table 1. All sequences are available on the NCBI repository Genbank. NCBI SRA sequence ID numbers and associated locality data are in Tables 1–3. All other materials and code needed to replicate the results of this study are deposited on Dryad (reviewer link: http://datadryad.org/share/LINK_NOT_FOR_PUBLICATION/v976swQBoss3sfFAB1tfDHBbNHHNssgunPkcecbVHlc).
