# Supplementary material for: Aquifer-Mediated Speciation in Cave-Adapted Fishes
Source: Integr Org Biol. 2026 May 18;8(1):obag021. doi: 10.1093/iob/obag021 (PMC13221641; doi:10.1093/iob/obag021)
Supplement: obag021_Supplemental_File [file obag021_supplemental_file.docx]

**Supplement To: Aquifer-mediated speciation in cave-adapted fishes**

**Authors:** Chase D. Brownstein,^1*†^ Gregory J. Watkins-Colwell,^2^ Maxime Policarpo^3,4^, Richard C. Harrington,^5^ Eva A. Hoffman,^6^ Didier Casane^7,8^ and Thomas J. Near^1,2^

**Affiliations:**

^1^Department of Ecology and Evolutionary Biology, Yale University, New Haven CT, USA

^2^Yale Peabody Museum, New Haven CT, USA

^3^Zoological Institute, Department of Environmental Sciences, University of Basel, Basel, Switzerland

^4^Evolution of Sensory and Physiological Systems, Max Planck Institute for Biological Intelligence, Martinsried, Germany

^5^Marine Resources Research Institute, South Carolina Department of Natural Resources

^6^Richard Gilder Graduate School, American Museum of Natural History, New York, NY, USA

^7^Université Paris-Saclay, CNRS, IRD, UMR Évolution, Génomes, Comportement et Écologie, 91198, Gif-sur-Yvette, France

^8^Université Paris Cité, UFR Sciences du Vivant, F-75013 Paris, France

*corresponding author, chase.brownstein@yale.edu

**The PDF file includes:**

Figs. S1 to S13

**Figure S1. Map showing the overlapping distributions of the three cavefish species.** Dots

marked with the letter S denote caves where *Typhlichthys styx* occurs in sympatry with *T.*

*subterraneus.*

**Figure S2. Phylogeny of *Typhlichthys* I.** Concatenated single partition maximum likelihood phylogeny of the 75% complete UCE matrix inferred using IQ-TREE2.

**Figure S3. Phylogeny of *Typhlichthys* II.** Concatenated multiple partition maximum likelihood phylogeny of the 75% complete UCE matrix inferred using IQ-TREE2.

**Figure S4. Phylogeny of *Typhlichthys* III.** ASTRAL-III species tree built using gene trees inferred from the 75% complete UCE matrix using IQ-TREE2.

**Figure S5. Phylogeny of *Typhlichthys* IV.** Maximum likelihood phylogeny of the ND2 matrix inferred using IQ-TREE2.

**Figure S6. Phylogeny of *Typhlichthys* V.** Bayesian maximum clade credibility phylogeny of the ND2 matrix inferred using MrBayes.

**Figure S7. Comparison of phylogenies of *Typhlichthys* inferred using UCEs and the ND2 mtDNA marker.** Numbers at nodes along ASTRAL phylogeny are coalescent support values. Numbers at nodes along ND2 maximum likelihood phylogeny are bootstrap support values.

**Figure S8. Heatmap of pairwise dissimilarity values across *Typhlichthys* individuals sampled for the ND2 marker.**

**Figure S9. Haplotype map of *Typhlichthys* individuals sampled for the ND2 marker.** Numbers along branches denote numbers of mutations separating haplotypes.

**Figure S10. Morphology of the retroarticular in species of *Typhlichthys*.** Arrows indicate the direction of the retroarticular in species of *Typhlichthys*.

**Figure S11. Bayesian node-dated phylogeny of *Amblyopsidae*.** Bayesian time-calibrated phylogeny inferred using BEAST2 using a node-dating approach. Bars at nodes indicate 95% highest posterior density intervals for divergence times.

**Figure S12. Curves showing relative numbers of biogeography events inferred using stochastic mapping in BioGeoBears.**

**Figure S13. Ages of *Typhlichthys* lineage divergences compared to ages of cave abandonments in the Cumberland River Drainage.**
